# Native oleaginous yeasts *Rhodotorula mucilaginosa* and *Solicoccozyma gelidoterrea*: a sustainable biotechnological alternative for lipid production with potential application in diets for farmed fish

**DOI:** 10.3389/ffunb.2026.1664434

**Published:** 2026-02-10

**Authors:** Paola Díaz-Navarrete, Luis Marileo, Hugo Madrid, Wladimir Mardones, David Correa Galeote, Nicolle Parra, Sebastián Dehnhardt-Amengual, Patricio Dantagnan

**Affiliations:** 1 Departamento de Ciencias Veterinarias y Salud Pública, Facultad de Recursos Naturales, Universidad Católica de Temuco, Temuco, Chile; 2 Escuela de Medicina Veterinaria, Facultad de Recursos Naturales y Medicina Veterinaria, Universidad Santo Tomás, Temuco, Chile; 3 Escuela de Tecnología Médica, Facultad de Medicina y Ciencias de la Salud, Universidad Mayor, Temuco, Chile; 4 Centro para la Resilencia, Adaptación y Mitigación (CReAM), Universidad Mayor, Temuco, Chile; 5 Instituto Milenio de Biología Integrativa, Santiago, Chile; 6 Departamento de Microbiología, Facultad de Farmacia, Universidad de Granada, Granada, Spain; 7 Departamento de Ciencias Agropecuarias y Acuícolas, Facultad de Recursos Naturales, Universidad Católica de Temuco, Temuco, Chile

**Keywords:** soil yeasts, volcanic soil yeast, lipids, PUFAs, *Rhodotorula mucilaginosa*, *Solicoccozyma gelidoterrea*, oleaginous yeast, aquaculture

## Abstract

**Introduction:**

The rapid global expansion of aquaculture has intensified the demand for sustainable and alternative lipid sources for fish feed formulations, driving interest in microbial platforms with specialized metabolic capabilities. Among these, oleaginous yeasts have emerged as promising candidates due to their ability to accumulate substantial intracellular lipid reserves and to modulate fatty acid composition in response to environmental and nutritional cues.

**Methods:**

In this study, the lipid production potential and physiological responses of two native yeast strains isolated from volcanic soils of southern Chile were investigated. The strains were identified by ITS sequencing as *Solicoccozyma gelidoterrea* (7C) and *Rhodotorula mucilaginosa* (Rho 6S). Growth kinetics, substrate utilization, and lipid accumulation were systematically evaluated under different carbon sources, carbon-to-nitrogen (C/N) ratios, and temperature regimes (7–25 °C). Response surface methodology was applied to determine the combined effects of nutritional and thermal factors on biomass production and lipid yield, while fatty acid composition was analyzed to elucidate lipid remodeling strategies.

**Results and Discussion:**

*R. mucilaginosa* exhibited pronounced metabolic versatility, characterized by higher maximum specific growth rates on alternative carbon sources such as xylose, sucrose, and raffinose. Under optimal conditions (25 °C and C/N 20), this strain achieved a lipid content of 30% and a biomass concentration of 2.54 g/L. In contrast, *S. gelidoterrea* displayed a distinct physiological profile associated with cold adaptation, reaching optimal lipid accumulation at 7 °C and C/N 20, with 26.6% lipid content and 2.11 g/L biomass. Increasing the C/N ratio to 90 significantly constrained lipid accumulation in both strains, highlighting the central role of nitrogen availability in regulating yeast lipid metabolism. Fatty acid profiling revealed clear species-specific lipid remodeling patterns: *R. mucilaginosa* produced a nutritionally favorable lipid profile enriched in mono and polyunsaturated fatty acids, reflected by high MUFA/SAFA and PUFA/SAFA ratios. In contrast, *S. gelidoterrea* exhibited a distinctive lipid profile dominated by monounsaturated fatty acids, particularly oleic acid, under nitrogen limited and low temperature conditions, and demonstrated the capacity to synthesize long chain polyunsaturated fatty acids under stress conditions, suggesting the activation of adaptive and stress responsive lipid metabolic pathways.

**Conclusion:**

This study provides the first evidence of lipid accumulation and fatty acid composition in *S. gelidoterrea* and puts into evidence contrasting lipid metabolic strategies among native oleaginous yeasts. Collectively, these findings contribute to a deeper understanding of fungal lipid physiology and environmental adaptation and support the potential of native yeast strains as sustainable lipid sources for functional foods and aquaculture nutrition.

## 1 Introduction

The sustained growth of aquaculture, mainly fish and shrimp, as a strategic sector for global food security has led to an increased demand for ingredients used in the formulation of balanced feeds, particularly those rich in high-quality lipids and proteins (Zlaugotne et al., 2022). Currently, fish oils and meals are key components in many aquafeed systems due to their high bioavailability of essential nutrients, such as long-chain polyunsaturated fatty acids (PUFAs), especially EPA and DHA (Sprague et al., 2017; Gasco et al., 2018; Parrish, 2024). However, the intensive exploitation of fishery resources has raised environmental, economic, and ethical concerns, highlighting the urgent need to identify alternative and sustainable lipid sources (Brunel et al., 2022). In this context, oleaginous yeasts have emerged as one of the most promising alternatives (Parrish, 2024). Several authors have reported lipid accumulation in yeasts (Blomqvist et al., 2018; Naveira-Pazos et al., 2023; Markuš et al., 2025) with the most promising species for lipid production including *Rhodosporidium toruloides, Lipomyces starkeyi, Lipomyces tetrasporus, Cutaneotrichosporon curvatum, Candida diddensiae, Metschnikowia reukaufii, Candida tropicalis, Solicoccozyma terricola, Naganishia albida, Papiliotrema laurentii, Rhodotorula glutinis, Rhodotorula graminis, Rhodotorula mucilaginosa, Apiotrichum domesticum, Trichosporon asahii, Tausonia pullulans, Yarrowia lipolytica, and Schwanniomyces etchellsii etchellsii* (Díaz-Navarrete et al., 2023; Nunes et al., 2024; Wusnah et al., 2023). Most of these yeasts accumulate lipids primarily in the cytoplasmic membrane, representing approximately 25% of the cell dry weight, in the form of triglycerides (TGs) (Småros et al., 2025), with maximum TG production typically occurring under nutrient-limiting conditions such as nitrogen or phosphorus deficiency, combined with an excess carbon source (Correa-Galeote et al., 2022). Among the species of interest, *Rhodotorula mucilaginosa* (Prabhu et al., 2019; Li et al., 2022; Tsai et al., 2022) and *Solicoccozyma* spp. (Filippucci et al., 2016) have stood out for their ability to produce microbial oils with lipid profiles comparable to those of vegetable oils and, in some cases, enriched in polyunsaturated fatty acids beneficial to animal health (Stefańska et al., 2018). These yeasts can be cultivated on media composed of agro-industrial residues (Aiello et al., 2021; Di Fidio et al., 2021), organic-rich wastewater (Wusnah et al., 2023), or food industry by-products, which not only reduces production costs but also provides added value in terms of sustainability and circular economy (Lopes da Silva et al., 2023; Pereira et al., 2023). Lipid accumulation in oleaginous yeasts is governed by a range of physiological, nutritional, and environmental factors (Wang et al., 2025). One of the most critical factors is the carbon/nitrogen (C/N) ratio, which, when elevated, promotes lipid accumulation by redirecting cellular metabolism toward lipogenesis under nitrogen-limited conditions (Zhou et al., 2021; Rosas-Paz et al., 2024). Additionally, the type of carbon source (Filippucci et al., 2016) and the availability of micronutrients such as phosphorus, sulfur, and magnesium directly influence lipogenic capacity. Particularly notable is the effect of phosphate limitation (Huang et al., 2018) which has been shown to enhance lipid accumulation in species such as *Yarrowia lipolytica*, although often at the expense of reducing total biomass (Carmon et al., 2024). The physicochemical conditions of the culture, such as pH and temperature, also modulate lipid biosynthesis (Salvador López et al., 2022). pH values between 5.0 and 6.5 are generally optimal for triacylglycerol accumulation in oleaginous yeasts (Zhou et al., 2021). Temperature, in turn, affects both growth and fatty acid composition: lower temperatures tend to favor higher unsaturation levels, whereas higher temperatures enhance biomass production but may reduce lipid content (Salvador López et al., 2022). Other critical factors include oxygen availability, which regulates the activity of key enzymes involved in lipogenesis, and the concentration of the carbon source, which must be maintained at appropriate levels to avoid inhibitory effects (Nunes et al., 2024). Understanding and controlling these parameters are essential for optimizing lipid production for industrial purposes, such as the manufacture of nutraceutical products (Li et al., 2022). From a nutritional perspective, lipids extracted from yeasts hold considerable potential as functional ingredients that can be incorporated into the diets of fish and crustaceans (Hatlen et al., 2012; Blomqvist et al., 2018; Emam et al., 2020; Brunel et al., 2022). However, the application of oleaginous yeasts in aquaculture is not limited to their lipid fraction, as they can be considered a source of other important compounds. Certain strains, such as *Rhodotorula* spp., in addition to their oleaginous potential (Li et al., 2022; Zhang et al., 2024), have demonstrated the ability to synthesize carotenoids such as β-carotene and torulene, antioxidant compounds that may strengthen the immune system of aquatic animals and enhance their resistance to pathogens (Brunel et al., 2022; Cheng and Yang, 2016; Li et al., 2022). Moreover, this biomass, rich in proteins, polysaccharides, and bioactive compounds, can also be used as protein or prebiotic sources, promoting intestinal health and supporting a more balanced intestinal microbiota in farmed (Ceseña et al., 2021). This integrated approach allows for the maximization of resource utilization and the development of multifunctional products, thereby supporting a more sustainable and efficient production model (Filippucci et al., 2016). Research indicates that yeasts such as *Lipomyces starkeyi* and *Rhodotorula toruloides* can effectively replace vegetable oils in fish diets without compromising growth performance or health in species such as Arctic char (*Salvelinus alpinus*) (Blomqvist et al., 2018). In the study conducted by Sanahuja et al. (2023) the effects of a diet supplemented with *Debaryomyces hansenii* in gilthead seabream (*Sparus aurata*) were evaluated by analyzing key performance indicators (KPIs) related to somatic growth and feed efficiency. Additionally, the study investigated transcriptomic profiles, histological organization of the anterior-mid intestine, and native microbiota composition to better understand how a diet including *D. hansenii* influences fish physiology. Dietary supplementation with yeast in juvenile gilthead seabream fed low fishmeal diets significantly improved somatic growth and feed efficiency (with a 12% increase), without adverse effects on intestinal health. Transcriptomic analysis revealed the modulation of key metabolic pathways, including protein, lipid, and nucleotide metabolism, along with enhanced antioxidant and immune responses. Similarly, in the study conducted by Brunel et al. (2022), the partial inclusion of biomass from the oleaginous yeast *Rhodotorula toruloides* as a substitute for vegetable oils in the diet of Arctic char (*Salvelinus alpinus*) was evaluated. The results indicated a significant increase in liver weight and hepatosomatic index in the experimental group, without notable differences in hepatic and muscle lipid content or histological alterations in the liver. Although the fatty acid profile in muscle remained comparable across groups, a higher proportion of monounsaturated fatty acids was observed in the liver of fish fed yeast biomass. In another study, Singh et al. (2024), assessed the effect of whole and autolyzed *Yarrowia lipolytica* on juvenile rainbow trout, reporting that dietary inclusion of 5% whole yeast enhanced intestinal immune gene expression, although no significant changes were observed in growth or overall microbiota composition. Undoubtedly, the nutritional characteristics and lipid profiles of these microbial oils have a direct impact on various aspects of animal health, which are determined by key lipid nutritional indices such as MUFA/SAFA, PUFA/SFA, and LA/ALA ratios, among others (Carvalho et al., 2022; Azcuy et al., 2025) and could eventually be incorporated into fish diets at low levels of inclusion, production technology allows it at a reasonable cost.

Our study aimed to enhance and evaluate lipid accumulation in the native yeasts *R. mucilaginosa* Rho 6S and *Solicoccozyma gelidoterrea* 7C, and to analyze their lipid profiles under different cultivation conditions.

## 2 Methodology

### 2.1 Microorganisms

Two native oleaginous yeast strains *R. mucilaginosa* Rho 6S and *S. gelidoterrea* 7C were isolated from the soil of the Araucanía Region at coordinates 38°42´08.1´´S 72°32´49.7W (North Campus UCT) and 39°21´32.9´´S 71°53´38.W¨W (Cerduo Sector at the Villarrica volcano slopes), respectively. These strains were molecularly identified and are registered in the GENBANK database (Rho 6S: PP209387.1.,7C: PV784691). The native yeast *R. mucilaginosa* Rho 6S has been preliminarily characterized and molecularly identified (Díaz-Navarrete et al., 2024).

### 2.2 Preparation of the inoculum and strain maintenance

The yeast was kept on solid medium at 4°C in Sabouraud Agar and replicated every 4 weeks to create fresh culture. The yeast culture was stored in a cryopreserver at −80°C after frozen in liquid nitrogen. The inoculum was prepared in liquid medium containing 2% glucose, 2% peptone, and 1% yeast extract, in a final volume of 100 mL. The medium was inoculated with a fresh culture of yeast cultured for 24 h with cells in exponential phase at a concentration of 1 × 10^6^ cells/mL. The cultures were placed in a reciprocal shaker at a temperature of 25°C and 150 rpm until the exponential phase was reached.

### 2.3 PCR amplification and yeast DNA sequencing

The general characterization of the strain 7C is described below: The partial amplification of the 26S gene of nuclear ribosomal DNA (rDNA) was amplified using primers ITS1 (5′ TCC GTA GGT GAA CCT GCG G 3’) and ITS4 (5´-TCC TCC GCT TAT TGA TAT GC-3´) (Amselem et al., 2011). The PCR reaction was carried out in 25 μL containing GoTaqR Green Master Mix (Taq DNA polymerase, dNTPs, MgCl2) (Promega, Madison, WI, USA) and 0.5 μL of the forward and reverse primers. Amplification of the ITS1-ITS4 fragment was performed under the following conditions: initial denaturation (2 min at 95°C), 30 cycles of denaturation (94°C for 1 min), annealing (40 s at 52°C), and extension (1 min at 72°C), followed by a final extension (10 min at 72°C) as elsewhere described (Díaz-Navarrete et al., 2023). 4 μL of the PCR product were analyzed on 1.5% TAE 1X agarose gels and visualized using a FOTO/UV21 Transilluminator Haverhill, MA, USA. A 100 bp DNA molecular weight marker (Thermo Fisher Scientific Inc., Waltham, MA, USA) was used. The DNA concentration was measured on a Nanodrop, Infinite M200. The amplified DNA was purified and sent for sequencing to Austral-Omics, Valdivia-Chile.

### 2.4 Molecular identification of yeast strains

A consensus ITS sequence was obtained from the complementary sequences of strain 7C using the SeqTrace software. A BLAST search (Altschul et al., 1990) was carried out in order to compare the sequence of the studied strain with those of fungal species currently represented in GenBank. ITS sequences of ex-type strains and other reference strains, representing all currently accepted species of *Solicoccozyma*, were retrieved for the phylogenetic study. DNA sequence alignments were made with the MUSCLE webserver (http://www.ebi.ac.uk/Tools/msa/muscle/) and then adjusted manually with a text editor. Phylogenetic analyses were performed with the maximum likelihood (ML) and neighbor-joining (NJ) methods with MEGA X (Kumar et al., 2018), using the best DNA substitution model chosen by that software. The statistical support for the groupings was assessed by bootstrap analysis of 1000 replicates. The ITS dataset and phylogenetic trees were deposited in Figshare with the DOI 10.6084/m9.figshare.29321501.

### 2.5 Effect of different carbon sources and stress compounds on cell growth

Kinetic growth parameters were obtained from growth curves of yeast strains cultivated under 24 different conditions. All compounds were purchased from Merck and prepared at standard working concentrations commonly used in yeast phenotypic profiling: Caffeine (0.5 mM), CuSO_4_ (0.2 mM), DTT (2 mM), ethanol at 8%, 9%, and 10% (v/v), fructose (2% w/v), G418 (200 µg/mL), galactose (2% w/v), glycerol (2% v/v), hydrogen peroxide (H_2_O_2_, 2 mM), KCl (0.5 M), lactose (2% w/v), maltodextrin (2% w/v), maltose (2% w/v), methanol (8% v/v), NaCl (1 M), p-coumaric acid (0.5 mM), raffinose (2% w/v), sucrose (2% w/v), SDS (0.01% w/v), sorbitol (1 M), and xylose (2% w/v). Initial pre-cultures were established by inoculating yeast strains into 96-well microplates containing 200 µL of 2% YNB-glucose medium, followed by incubation for 24 h at 20°C for strain 7C and 25°C for strain Rho 6S. The reference strain used was *Saccharomyces cerevisiae* BY4741. After pre-growth, 5 µL of each culture were transferred into 195 µL of the corresponding test medium. Cultures were incubated using a Cytation 3 system equipped with a BioStack 4 (BioTek, USA), with optical density at 600 nm (OD_600_) recorded every hour over a 48-hour period. Growth parameters were calculated using the “gcplyr” package in R, and principal component analysis (PCA) was performed with a custom R script utilizing the prcomp function from the base stats package. Additionally, a heatmap was generated based on the maximal specific growth rate (µ_max_) of the yeast strains *R. mucilaginosa* (Rho 6S) and *S. gelidoterrea* (7C) under different carbon sources and stress-inducing compounds. The color scale represents µ_max_ values, where deep red indicates high growth, blue represents no or negative growth, and pale reddish tones reflect intermediate levels. Hierarchical clustering was applied to highlight similarities in growth patterns across experimental conditions.

### 2.6 Effect of temperature and (R C/N) on the accumulation of fatty acids in native yeasts

The native yeast strains (Rho 6S and 7C) were evaluated in batch culture under different incubation temperatures (Abaza et al., 2021) and varying carbon-to-nitrogen (C/N) ratios. The basal culture medium (BCM) contained 66.6 g/L glucose, 0.4 g/L MgSO_4_·7H_2_O, 2 g/L KH_2_PO_4_, 0.003 g/L MnSO_4_·H_2_O, 0.0001 g/L CuSO_4_·5H_2_O, 1.5 g/L yeast extract, and varying concentrations of (NH_4_)_2_SO_4_ (based on C/N ratio). For the calculation of the C/N ratio, it was considered that glucose contributes 0.4 g of carbon per gram of glucose, and ammonium sulfate contributes 0.2121 g of nitrogen per gram of (NH_4_)_2_SO_4_. The cultures were performed in 1000 mL Erlenmeyer flasks with a working volume of 600 mL, incubated at 200 rpm and pH 5.5, under appropriate temperatures for each strain (25°C for Rho 6S; 20°C for 7C). Cultivation was carried out in a reciprocal shaker. The inoculum size was 10% v/v (10_8_ cells/mL in exponential phase). Flasks were sterilized by autoclaving at 121°C, 1 atm for 15 minutes. Experiments were designed and conducted according to a Central Composite Design for Response Surface Methodology (RSM-CCD), considering two factors: C/N ratio (20–90) and temperature shift (7, 16, or optimal for each strain), by using Design-Expert^®^ software, version 12 (Stat-Ease Inc., Minneapolis, MN, USA). Cultures were initially grown at their optimal temperatures (25°C for Rho 6S and 20°C for 7C). After reaching the end of the exponential phase (48 h for Rho 6S and 72 h for 7C), the temperature was either reduced to 7°C or 16°C, or maintained at the optimal level, depending on the experimental condition. The following response variables were measured: biomass (g dry weight/L), specific productivity of total lipids (mg/g dry biomass), total lipid content, lipid profile (MUFA: Monounsaturated Fatty Acids, PUFA: Polyunsaturated Fatty Acids, SAFA: Saturated Fatty Acids, AL: Linoleic Acid (C18:2 ω6)., ALA: Alpha-Linolenic Acid (C18:3 ω3)), nutritional lipid indices: (LA/ALA (Linoleic Acid/Alpha-Linolenic Acid); MUFA/SAFA (MonoUnsaturated Fatty Acids/Saturated Fatty Acids), PUFA/SAFA (Polyunsaturated Fatty Acids/Saturated Fatty Acids).

### 2.7 Analytical methods

#### 2.7.1 Quantification of cell biomass (yeast)

Post-culture yield of yeast cell biomass will be determined after centrifuging in a previously weighed thimble (3000× g, 10 min, 4°C). Centrifuged cell biomass will be dried at a temperature of 80°C (WTC Binder oven) for 24 h to constant weight. Biomass yield will be expressed in grams of yeast dry weight (g d.w.) per litre of culture medium (g d.w.L^−1^ of medium).

#### 2.7.2 Fatty acid composition

Yeast biomass samples were analyzed for their fatty acid (FA) composition using gas chromatography (GC). Prior to lipid extraction, samples were disrupted by vigorous agitation with acid-washed glass beads (0.5 mm diameter) to ensure complete cell lysis, and moisture content was determined gravimetrically by drying aliquots at 105°C to constant weight, allowing data normalization on a dry weight basis. Homogenization was then performed using a T10 Basic Ultra-Turrax disperser (IKA, Staufen, Germany). Lipids were extracted following the (Breil et al., 2017) using a chloroform/methanol solution (2:1, v/v), with heptadecanoic acid (C17:0) added as an internal standard to monitor recovery and enable quantification. The extracted lipids were then methylated to fatty acid methyl esters (FAMEs) using 14% boron trifluoride (BF_3_) in hexane, following (Morrison and Smith, 1964). FAMEs were analyzed using a gas chromatograph (HP6890, Hewlett Packard, Palo Alto, USA) equipped with a flame ionization detector (FID) and helium as the carrier gas. Separation was achieved with a Supelco SP2380 capillary column (30 m × 0.25 mm i.d. × 0.20 μm film thickness), with injector and detector temperatures set at 220°C and 200°C, respectively. The oven temperature was programmed as follows: initial temperature of 60°C held for 1 minute, increased at 4°C/min to 204°C, followed by a 2°C/min increase to 240°C, and held at this final temperature for 2 minutes. Fatty acids were identified by comparison with a 37-component FAME standard mix (Supelco CRM47885, Sigma-Aldrich, St. Louis, USA) and quantified using HP ChemStation software (Agilent Technologies), based on calibration curves prepared from external standards. Analytical precision was verified by maintaining a coefficient of variation (CV) below 5% across replicate injections. Results were expressed as the percentage of peak area relative to the total identified fatty acids.

#### 2.7.3 Glucose content

Glucose concentration was measured by the 3,5-dinitrosalicylic acid (DNS) colorimetric method at 540 nm in which 3,5-DNS in alkaline solution is reduced to 3-amino-5-nitrosalicylic acid by reducing. Standard curve of glucose was prepared and used to estimate the concentration of the unknown reducing saccharide concentration (Sokač et al., 2023).

### 2.8 Statistical analysis

The differences in biomass production, total lipid content, fatty acid fractions (SAFA, MUFA, PUFA), and specific fatty acids (LA, ALA) under different temperature shock and C/N ratio conditions were assayed. Data normality was evaluated using the Shapiro–Wilk test, while homogeneity of variance was assessed with Levene’s test. As some variables did not meet the assumptions of normality and homoscedasticity, the non-parametric Kruskal–Wallis test was applied to detect overall differences. Subsequently, the Games–Howell multiple comparison test was used to assess intra-group differences, i.e., between the different C/N ratios evaluated within each temperature condition. This approach enabled precise identification of nutritional conditions that significantly influenced each specific temperature regime. All statistical analyses were performed using IBM SPSS Statistics software, version 31.0.0.0. Descriptive statistics were obtained for all response variables (biomass, total lipids, and fatty acid fractions: SAFA, MUFA, PUFA, ARA, LA, and ALA) under each temperature (25, 16, and 7°C) and C:N ratio (20, 55, and 90). To evaluate the effect of the C:N ratio within each temperature, a robust one-way ANOVA based on 20% trimmed means was applied using bootstrap resampling (599 iterations) to estimate p-values and confidence intervals. Effect sizes were calculated to quantify the magnitude of differences between treatments. The analysis was conducted in Jamovi software (version 2.6.45.0) at a significance level of p < 0.05. Results are presented in **Supplementary Tables TS4–TS7** of the **Supplementary Material**.

## 3 Results

### 3.1 Molecular identification

**Figure 1:**
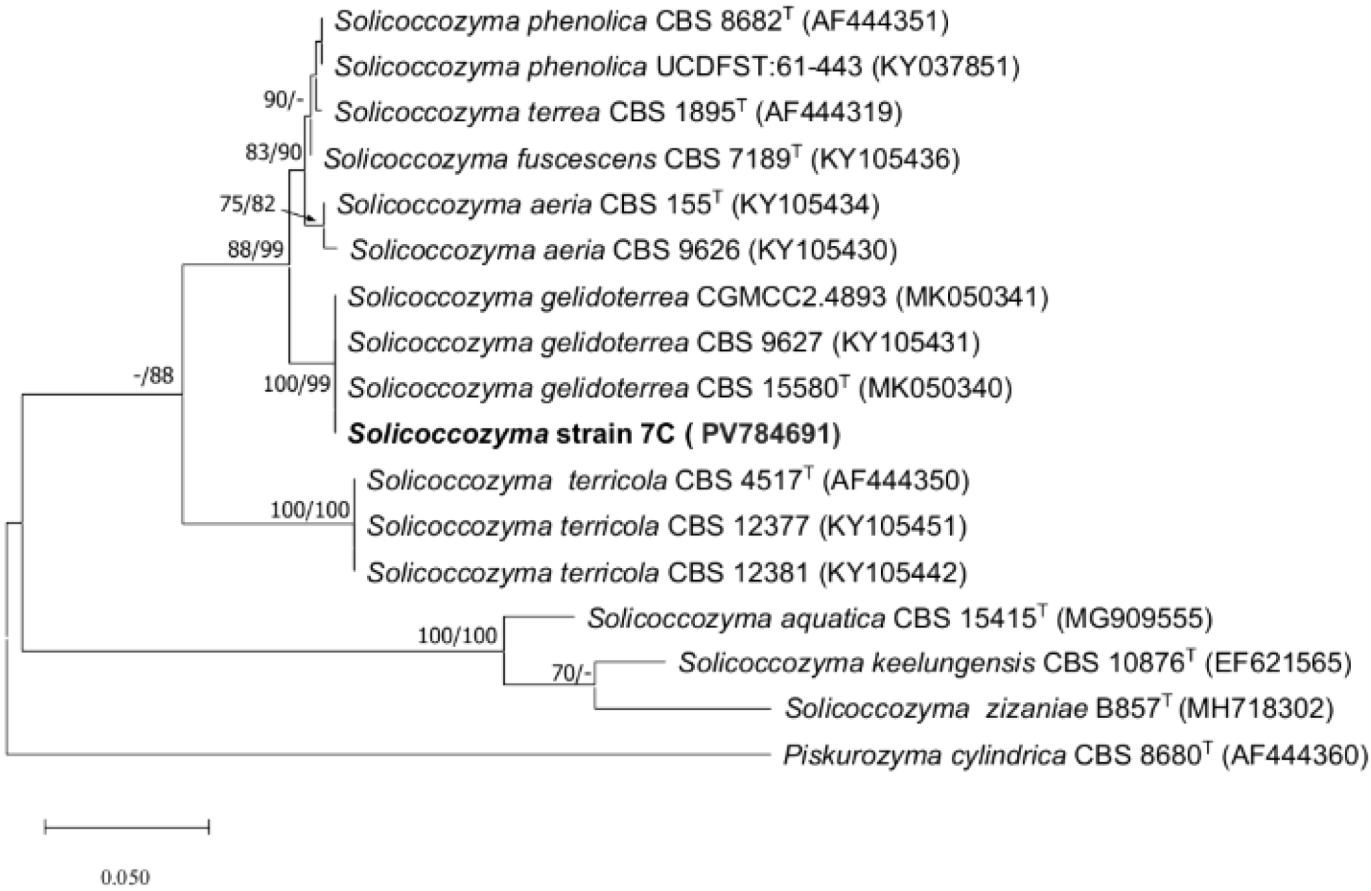
Maximum likelihood (ML) tree constructed with the ITS sequence of strain 7C and related species of *Solicoccozyma*. Branch lengths are proportional to distance. Bootstrap values ≥70% obtained in ML and neighbor-joining analyses, respectively, are shown near the internodes. T, ex-type strain. GenBank accession numbers of ITS sequences are given in parenthesis after strain numbers. *Piskurozyma cylindrica* was used as outgroup.

The BLAST search with the ITS sequence of strain 7C shown that, the closest matches were strains of *S. gelidoterrea*, such as CBS 15580 (ex-type strain, GenBank MK050340, identities 559/560, 99.82%, 1 gap), CBS 9627 (GenBank KY105431, identities 559/560, 99.82%, 1 gap), and CGMCC2.4893 (MK050341, identities 559/570, 98.07%, 11 gaps). Other close matches included *S. fuscescens* CBS 7189 (ex-type strain, GenBank KY105436, identities 540/564, 96%, 6 gaps), *S. phenolica* CBS 8682 (ex-type strain, GenBank AF444351, identities 540/565, 95.58%, 7 gaps), and *S. terrea* CBS 1895 (ex-type strain, GenBank AF444319, identities 539/564, 95.57%, 6 gaps). In phylogenetic analyses based on ITS sequences of all currently accepted *Solicoccozyma* species, strain 7C grouped with three strains of *S. gelidoterrea*, including the ex-type strain, forming a clade with 100% and 99% bootstrap support in the ML and NJ trees, respectively (**Figure 1**). DNA sequence comparisons and phylogeny reconstructions supported the identification of strain 7C as *S. gelidoterrea* 7C.

### 3.2 Analysis of the response profile to carbon sources and stressors in yeast strains

**Figure 2:**
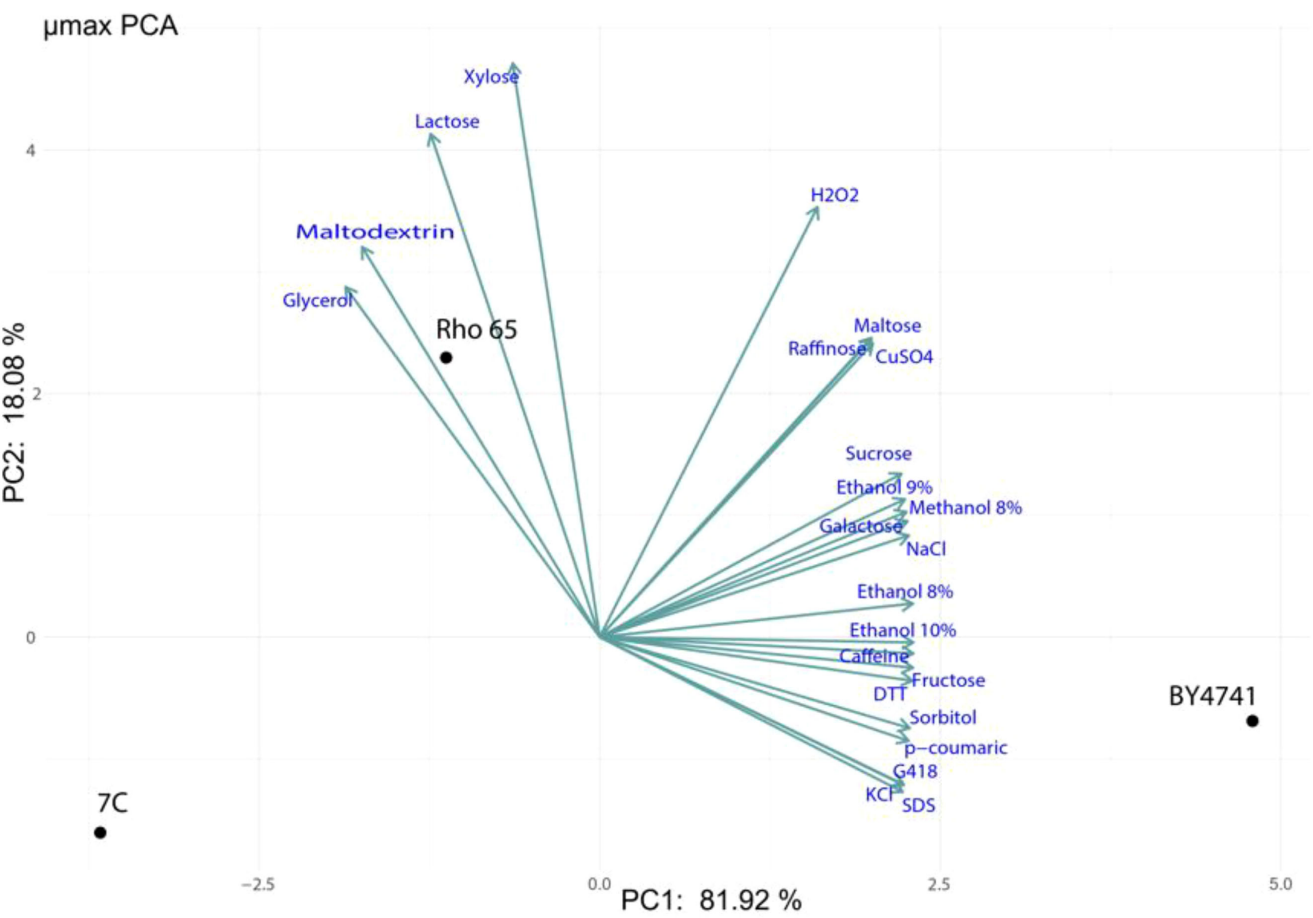
Principal component analysis (PCA) of the yeast strains *R. mucilaginosa* Rho 6S and *S. geloditerrea* 7C, based on the variation in carbon source and the presence of stress-inducing compounds. The PCA analysis was performed using the prcomp package in R. The PC1 explain the 81.92% of the variance and the PC2 explain the 18.08%.

The 7C, Rho 6S, and *S. cerevisiae* BY4741 (reference strain) strains were cultured in YNB media supplemented with different carbon sources and different stressors (Caffeine, CuSO_4_, DTT, Ethanol 8%, Ethanol 9%, Ethanol 10%, Fructose, Sucrose, G418, Galactose, Glycerol, H_2_O_2_, KCl, Lactose, Maltodextrin, Maltose, Methanol 8%, NaCl, p-coumaric, Raffinose, SDS, Sorbitol, Xylose), from the growth curves (OD 600 nm), the maximum specific growth rate was calculated (**Supplementary Table S1**). From these data, a principal component analysis (PCA) was performed using the µmax values obtained for three yeast strains: 7C, Rho 6S, and BY4741 (**Figure 2**). The first two principal components explained 100% of the total variance in the dataset, with PC1 accounting for 81.34% and PC2 for 18.66%. The PCA plot revealed a clear separation among the three strains. *S. cerevisiae* BY4741 clustered separately along the positive side of PC1, while Rho 6S was positioned on the opposite side. Strain 7C appeared in an intermediate position, slightly shifted along PC2. Interestingly, Rho 6S possesses a superior µmax in comparison with 7C when it is grown in Xylose, Sucrose, and Raffinose as a unique carbon source. These results suggest substantial phenotypic divergence in growth performance among the strains across the tested conditions.

**Figure 3:**
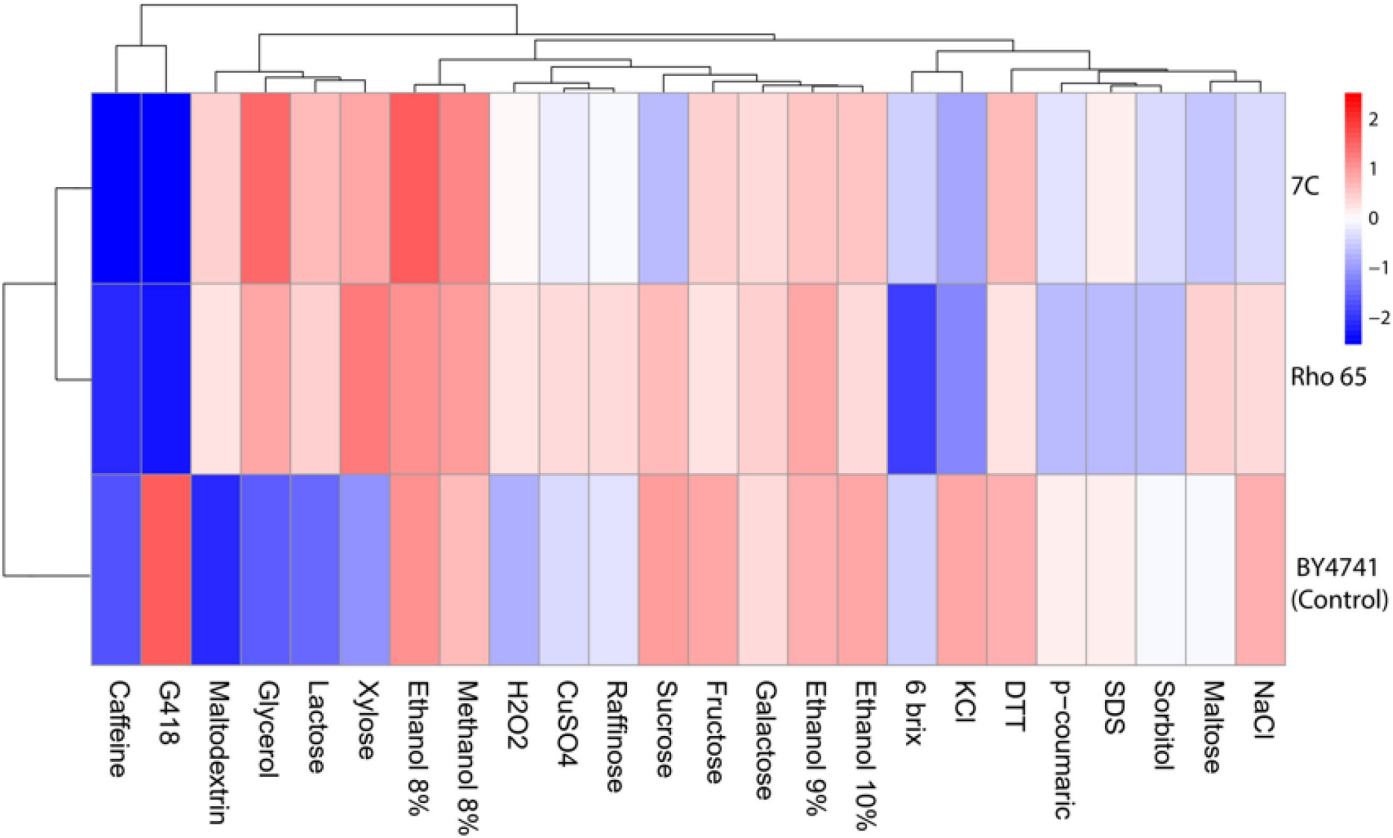
Heatmap of the maximal specific growth rate (µ_max_) of the yeast strains *R. mucilaginosa* (Rho 6S) and *S. gelidoterrea* (7C) under different carbon sources and stress-inducing compounds. The *S. cerevisiae* BY4741 laboratory stain was used as control. The color scale represents µ_max_ values, where deep red indicates high growth, blue represents negative or no growth, and pale tones correspond to intermediate values. Hierarchical clustering highlights similarities in growth patterns across experimental conditions. The heatmap and hierarchical analysis was performed using pheatmap package in r.

The heatmap representing the maximum specific growth rate (µ_max_) under different conditions reveals metabolic differentiation and stress tolerance between the yeast strains *R. mucilaginosa* (Rho 6S) and *S. gelidoterrea* (7C) (**Figure 3**). Strain Rho 6S exhibited high growth (intense red shades) on xylose, sucrose, and raffinose, indicating efficient assimilation of pentose and disaccharide sugars, which suggests strong potential for bioprocesses involving lignocellulosic waste. It also showed adequate tolerance to ethanol (Ethanol 8–10%) and hydrogen peroxide (H_2_O_2_), reflecting resistance to oxidative and ethanol-induced stress. In contrast, strain 7C demonstrated greater tolerance to caffeine and G418 (an aminoglycoside antibiotic), indicating adaptation to challenging growth conditions. It also showed intermediate performance on maltodextrin and methanol, suggesting moderate metabolic flexibility toward alternative carbon sources. Both strains maintained acceptable growth under stress inducing compounds such as CuSO_4_ (metal stress), DTT (redox stress), KCl and NaCl (osmotic stress), and SDS (an anionic detergent), although Rho 6S was more sensitive to caffeine and G418 compared to 7C. Notably, the 6 brix must condition (high concentration of soluble sugars) was particularly restrictive for Rho 6S. The laboratory control strain (*S. cerevisiae* BY4741) exhibited lower and more limited growth patterns (pale or bluish tones), confirming the superior adaptability of both wild isolates. Hierarchical clustering grouped conditions based on physiological similarities, distinguishing clusters of osmotic, oxidative, and fermentable compounds, thereby providing key insights for selecting cultivation conditions in industrial processes. This differential profile suggests that both strains possess specialized metabolic pathways and stress tolerance mechanisms that could be leveraged for specific biotechnological applications, such as fermentation using waste-derived media or biomass production under adverse conditions.

### 3.3 Response surface analysis in yeast strains Rho 6S and 7C

**Figure 4:**
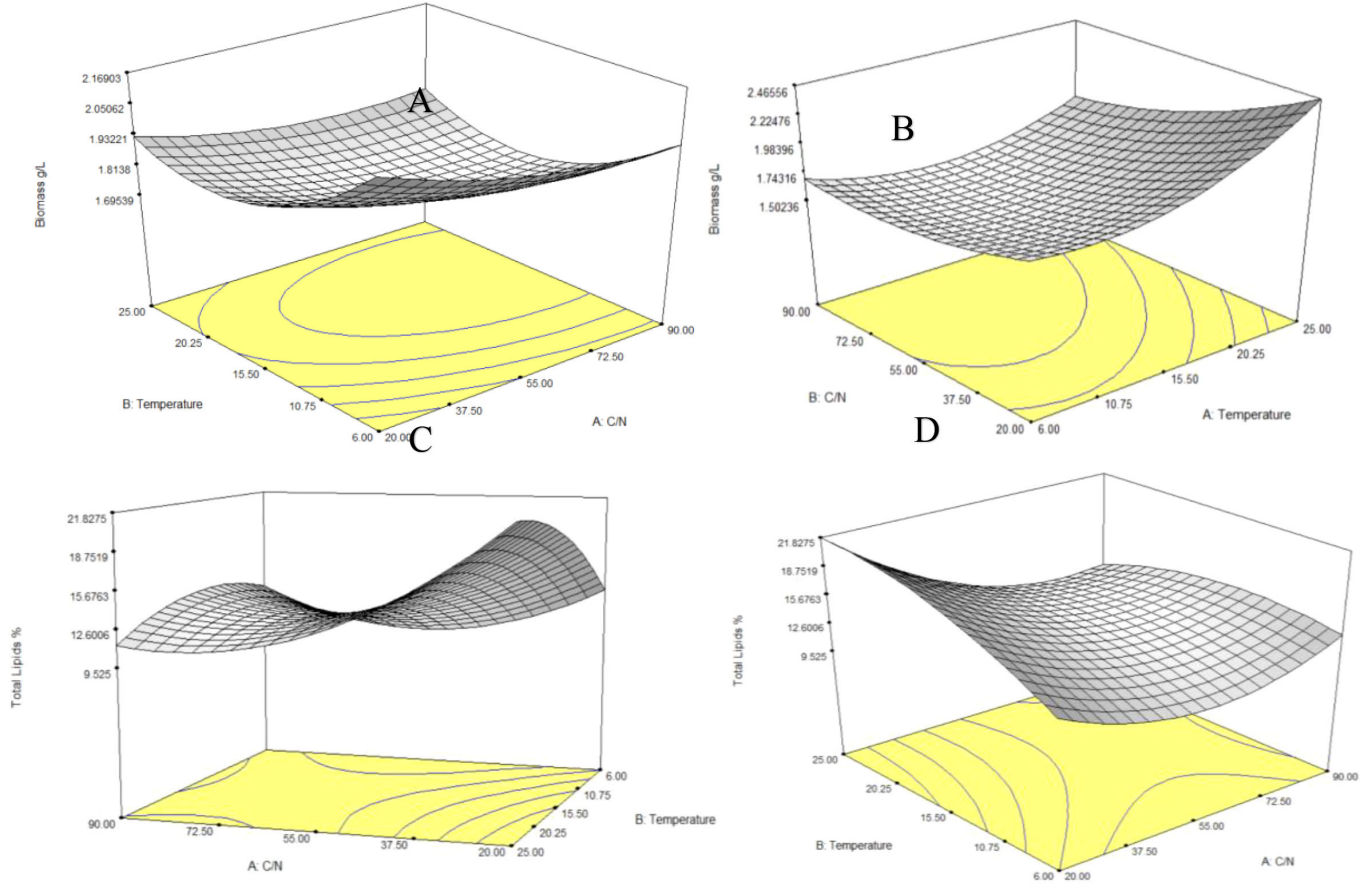
Response surface plots showing the effectt of temperature and the C/N ratio on: **(A)** cell growth (dry weight, g/L) of *R. mucilaginosa* Rho 6S; **(B)** lipid accumulation (% lipids, expressed as g of lipids × 100/total lipids) in *R. mucilaginosa* Rho 6S; **(C)** cell growth (dry weight, g/L) of *S. gelidoterrea* 7C; and **(D)** lipid accumulation (% lipids, expressed as g of lipids × 100/total lipids) in *Solicoccozyma gelidoterrea* 7C.

According to the findings, the optimal conditions for lipid accumulation in *R. mucilaginosa* (Rho 6S) were attained at a carbon-to-nitrogen (C/N) ratio of 20 and a temperature of 25°C. Under these conditions, maximum lipid levels were obtained, reaching 30.0%, along with a biomass of 2.54 g/L. At an intermediate temperature (16°C), high lipid levels were also observed at C/N 20 and 55 (up to 26.47% and 22.27%, respectively), but these values decreased significantly under C/N 90 conditions, reaching minimum values between 8.13% and 8.40%, consistent with the lower relative carbon availability for lipid biosynthesis. At low temperature (6°C), moderate lipid accumulation was observed at C/N 20 (22.0% and 20.1%), followed by a progressive decrease as the C/N ratio increased, reaching minimum levels at C/N 90 (11.04% and 13.07%). Nevertheless, biomass levels remained acceptable (>1.7 g/L under most conditions), highlighting the strain’s ability to grow at low temperatures. Based on these results, it can be inferred that strain Rho 6S exhibits maximum lipid accumulation under conditions of high carbon availability (C/N 20) and optimal temperature (25°C). Conversely, reduced carbon availability (C/N 90) negatively affects both biomass production and lipid content, regardless of the temperature (**Figures 4A, B**).

For *S. gelidoterrea* (7C), the highest lipid accumulation (26.60%) was observed at 7°C and C/N 20, accompanied by high biomass levels (2.11 g/L), suggesting that this strain responds favorably to cold stress conditions combined with carbon excess. At the same temperature (7°C) but under C/N 90, moderate lipid values were observed (12.07–14.00%). At intermediate temperatures (16°C), the results were more variable. Under C/N 20, lipid content reached up to 20.87%, whereas at C/N 55 and 90, a broad dispersion was recorded (5.13–17.73%), indicating lower stability in lipid accumulation under these conditions. However, notable biomass values (>1.7 g/L) remained relatively constant. At 25°C, a general reduction in lipid content was evident, particularly under C/N 55 conditions (6.80–15.40%), potentially reflecting reduced lipogenic efficiency at higher temperatures. Under C/N 20, lipid content ranged from 19.47% to 22.13%, confirming a preference for carbon-rich conditions. These findings suggest that strain 7C displays its highest lipid accumulation capacity at low temperatures (7°C) under carbon-rich conditions (C/N 20) (**Figures 4C, D**).

### 3.4 Lipid profile analysis in rho 6S and 7C strains

**Figure 5:**
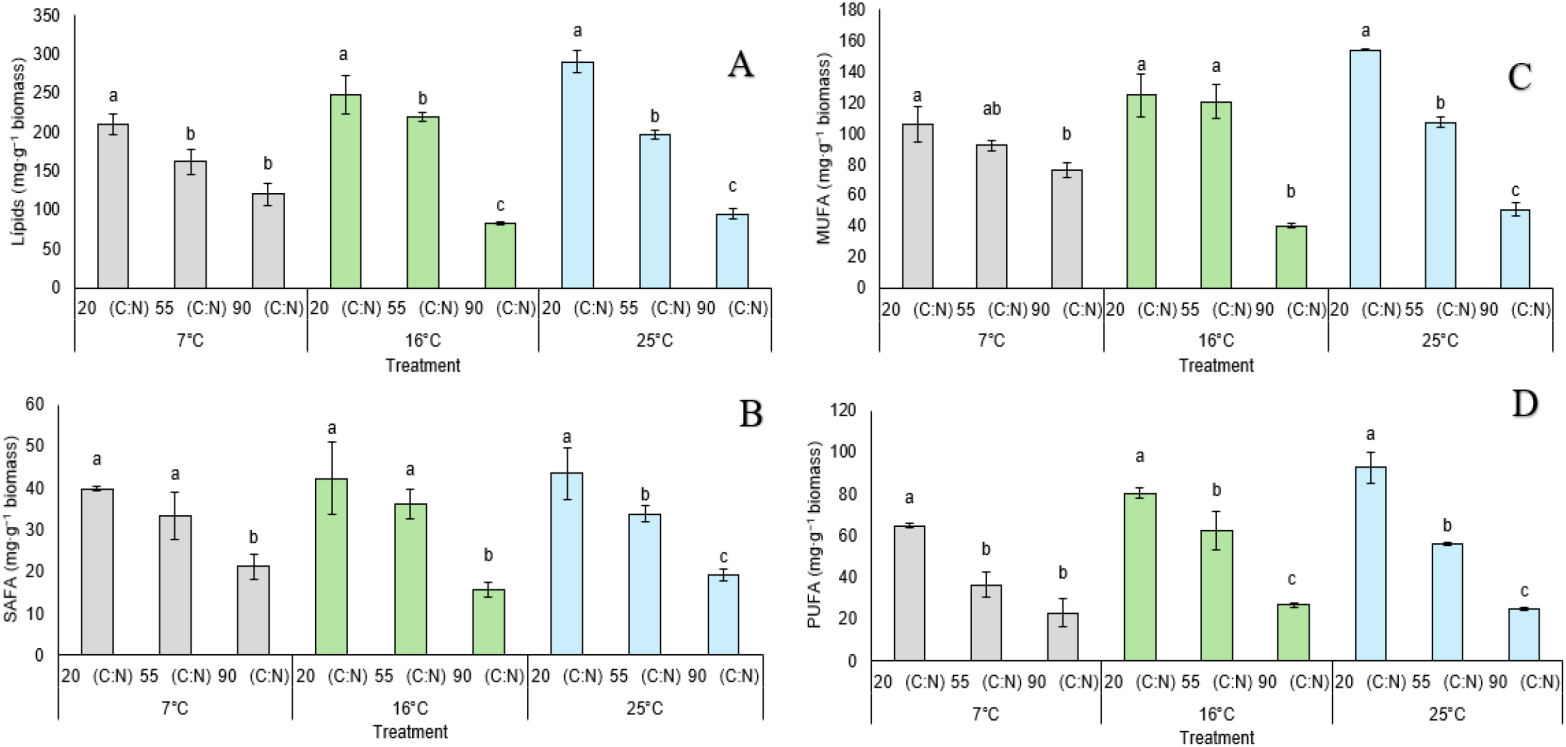
Total lipid production and fatty acid composition in *R. mucilaginosa* Rho 6S cultivated at 25°C with a temperature shift applied at 48 hours (to 7°C, 16°C, and 25°C) and carbon/nitrogen (C/N) ratios of 20, 55, and 90. **(A)** Total lipids (mg·g^−1^ biomass); **(B)** Saturated fatty acids (SAFA, mg·g^−1^ biomass); **(C)** Monounsaturated fatty acids (MUFA, mg·g^−1^ biomass); **(D)** Polyunsaturated fatty acids (PUFA, mg·g^−1^ biomass). Bars represent mean ± standard deviation. Different letters indicate statistically significant differences within each temperature group according to Games-Howell *post hoc* test (p < 0.05).

To understand how culture conditions influence the lipid profile of Rho 6S, a quantitative analysis was conducted on total lipid production, as well as the proportion of saturated (SAFA), monounsaturated (MUFA), and polyunsaturated (PUFA) fatty acids, in response to the imposed conditions. At 7°C, a progressive decrease in total lipids was observed (**Figure 5A**) with increasing C/N ratios, reaching values of 210.5 ± 13.4 mg/g at C/N 20, 162.0 ± 15.6 mg/g at C/N 55, and 120.6 ± 14.3 mg/g at C/N 90, with significant differences among all treatments. This pattern suggests that at low temperatures, reduced nitrogen availability leads to lower lipid accumulation. At 16°C, the highest values were recorded at C/N 20 (247.4 ± 24.5 mg/g), significantly decreasing at C/N 55 (219.0 ± 5.2 mg/g), and sharply declining at C/N 90 (82.7 ± 1.9 mg/g), indicating that nitrogen limitation severely restricts lipogenesis at this temperature. At 25°C, the highest lipid contents of the study were achieved, with 290.0 ± 14.1 mg/g at C/N 20 and significant reductions to 196.8 ± 5.8 mg/g (C/N 55) and 95.0 ± 7.1 mg/g (C/N 90), suggesting that a 16°C shock condition favors lipid accumulation, especially under low C/N ratios. SAFA contents (**Figure 5B**) exhibited greater sensitivity compared to other lipid fractions. At 7°C, the highest value was observed at C/N = 20 (39.9 ± 0.48 mg·g^−1^), decreasing to 33.4 ± 5.73 mg·g^−1^ at C/N = 55 and 21.2 ± 2.96 mg·g^−1^ at C/N = 90. At 16°C, a similar trend was maintained, with values of 42.4 ± 8.69, 36.2 ± 3.43, and 15.7 ± 1.65 mg·g^−1^, respectively. At 25°C, the optimal condition was again C/N = 20 (43.5 ± 6.28 mg·g^−1^), decreasing with higher C/N ratios. MUFA content (**Figure 5C**) showed the highest values among all lipid fractions. At 7°C, it ranged from 105.8 ± 11.83 mg·g^−1^ (C/N = 20) to 76.3 ± 4.62 mg·g^−1^ (C/N = 90). At 16°C, values increased to 124.6 ± 13.66 (C/N = 20) and 120.3 ± 11.05 mg·g^−1^ (C/N = 55), but dropped significantly at C/N = 90 (40.2 ± 1.46 mg·g^−1^). At 25°C, the MUFA fraction peaked at 153.8 ± 0.46 mg·g^−1^ (C/N = 20), progressively declining to 107.1 ± 3.14 (C/N = 55) and 50.9 ± 4.65 mg·g^−1^ (C/N = 90). The PUFA fraction (**Figure 5D**) showed high sensitivity to experimental conditions. At 7°C, values decreased with increasing C/N ratios: 64.8 ± 1.13 (C/N = 20), 36.5 ± 6.25 (C/N = 55), and 23.1 ± 6.77 mg·g^−1^ (C/N = 90). At 16°C, the highest accumulation was found at C/N = 20 (80.4 ± 2.18 mg·g^−1^), dropping sharply to 26.7 ± 1.20 mg·g^−1^ at C/N = 90. At 25°C, the same pattern was observed, with a maximum of 92.7 ± 7.41 mg·g^−1^ at C/N = 20 and a minimum of 24.9 ± 0.91 mg·g^−1^ at C/N = 90. The observed trend suggests that both cultivation temperature and nitrogen availability exert a significant influence on the strain’s ability to accumulate polyunsaturated lipids, with 25°C and a C/N ratio of 20 constituting the most favorable condition for enhanced lipid accumulation (**Supplementary Table S2**).

**Figure 6:**
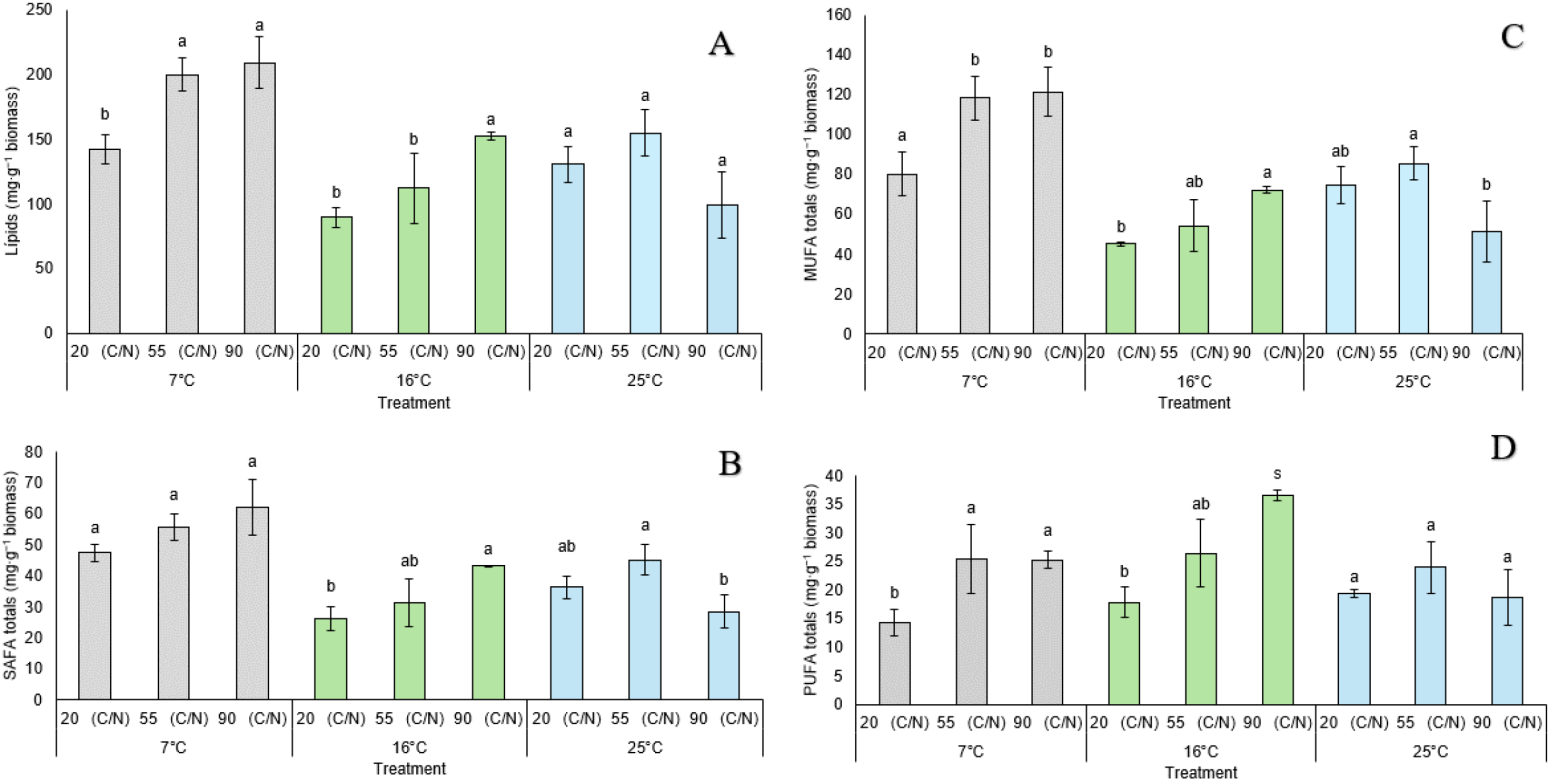
Total lipid production and fatty acid composition in *S. gelidoterrea* strain 7C cultured at 25°C, with a temperature shift applied at 48 hours (to 7°C, 16°C, or maintained at 25°C), under carbon/nitrogen (C/N) ratios of 20, 55, and 90. **(A)** Total lipids (mg·g^−1^ biomass); **(B)** Saturated fatty acids (SAFA, mg·g^−1^ biomass); **(C)** Monounsaturated fatty acids (MUFA, mg·g^−1^ biomass); **(D)** Polyunsaturated fatty acids (PUFA, mg·g^−1^ biomass). Bars represent mean values ± standard deviation. Different letters indicate statistically significant differences within each temperature group based on the Games-Howell *post hoc* test (p < 0.05).

The effect of temperature and C/N ratio on the lipid profile of *S. gelidoterrea* strain 7C was also evaluated by quantifying total lipids and the individual SAFA, MUFA, and PUFA fractions under different culture conditions (**Figure 6**). SAFA content (**Figure 6A**) varied notably across conditions. At 7°C, the highest levels were observed at C/N = 55 (55.8 ± 4.18 mg·g^−1^) and C/N = 90 (62.1 ± 9.17 mg·g^−1^), both significantly greater than at C/N = 20 (47.4 ± 2.66 mg·g^−1^). At 16°C, SAFA content was lowest at C/N = 20 (26.2 ± 3.89 mg·g^−1^) and increased progressively at C/N = 55 (31.2 ± 7.79 mg·g^−1^) and C/N = 90 (43.1 ± 0.26 mg·g^−1^), though the differences between the latter two were not statistically significant. At 25°C, no significant differences were detected among treatments; however, a slight decrease was noted at C/N = 90 (28.5 ± 5.38 mg·g^−1^). For the MUFA fraction (**Figure 6C**), at 7°C, significantly higher accumulation was observed at C/N = 55 (118.4 ± 10.93 mg·g^−1^) and C/N = 90 (121.4 ± 12.35 mg·g^−1^) compared to C/N = 20 (80.3 ± 10.99 mg·g^−1^). At 16°C, MUFA content ranged from 45.3 ± 1.01 mg·g^−1^ (C/N = 20) to 72.3 ± 1.62 mg·g^−1^ (C/N = 90), with C/N = 55 (54.2 ± 12.92 mg·g^−1^) showing intermediate values. At 25°C, MUFA levels were more consistent across treatments, with no significant differences detected. PUFA levels (**Figure 6D**) showed greater variability across conditions. At 7°C, the lowest content was recorded at C/N = 20 (14.4 ± 2.34 mg·g^−1^), while significantly higher levels were found at C/N = 55 (25.5 ± 5.99 mg·g^−1^) and C/N = 90 (25.3 ± 1.51 mg·g^−1^). At 16°C, PUFA content peaked at C/N = 90 (36.6 ± 0.94 mg·g^−1^), significantly surpassing values at C/N = 55 (26.5 ± 5.95 mg·g^−1^) and C/N = 20 (17.8 ± 2.67 mg·g^−1^), indicating that nitrogen limitation at this intermediate temperature enhances polyunsaturated lipid accumulation. At 25°C, no significant differences were observed, with moderate PUFA levels ranging from 18.7 to 24.0 mg·g^−1^. These findings highlight the sensitivity of the lipid profile in *S. gelidoterrea* strain 7C to culture conditions, with 16°C combined with a high C/N ratio emerging as particularly favorable for PUFA synthesis.

### 3.5 Nutritional index of yeast oil (MUFA/SFA, PUFA/SFA y LA/ALA)

**Figure 7:**
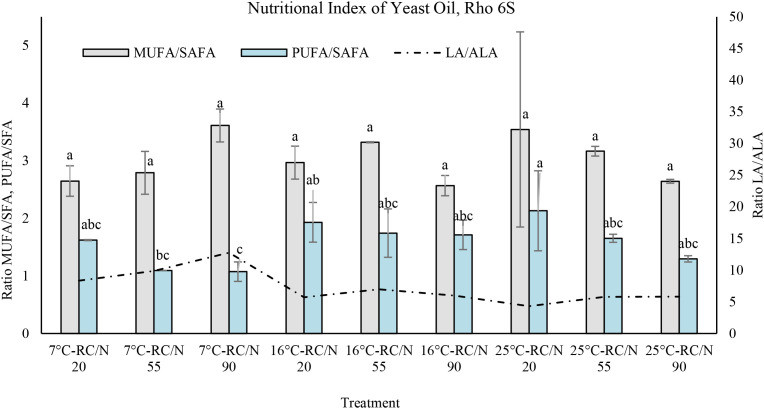
Nutritional indices of *R. mucilaginosa* Rho 6S oil under different temperature conditions (thermal shift to 7°C, 16°C, or maintained at 25°C) and carbon/nitrogen (C/N) ratios of 20, 55, and 90. Shown are the MUFA/SAFA and PUFA/SAFA ratios (left y-axis), and the LA/ALA ratio (right y-axis). Different letters indicate statistically significant differences (p < 0.05).

In *R. mucilaginosa* Rho 6S, significant variations were observed in the MUFA/SAFA, PUFA/SAFA, and LA/ALA ratios under different temperature and C/N ratio conditions, with relevant implications for the functional quality of the lipids produced (**Figure 7**). The MUFA/SAFA ratio reached its highest value at 7°C–C/N 90 (3.61 ± 0.29), indicating a higher proportion of monounsaturated fatty acids under cold and nitrogen-limited conditions; however, the differences were not statistically significant. The PUFA/SAFA ratio was highest at 25°C–C/N 20 (2.13 ± 0.69), suggesting that warm temperatures promote polyunsaturated fatty acid accumulation, albeit with greater variability. The LA/ALA ratio peaked at 7°C–C/N 90 (12.76 ± 1.02), and decreased markedly at 25°C–C/N 20 (4.31 ± 1.40), indicating a potential imbalance in the omega-6/omega-3 ratio under these conditions.

**Figure 8:**
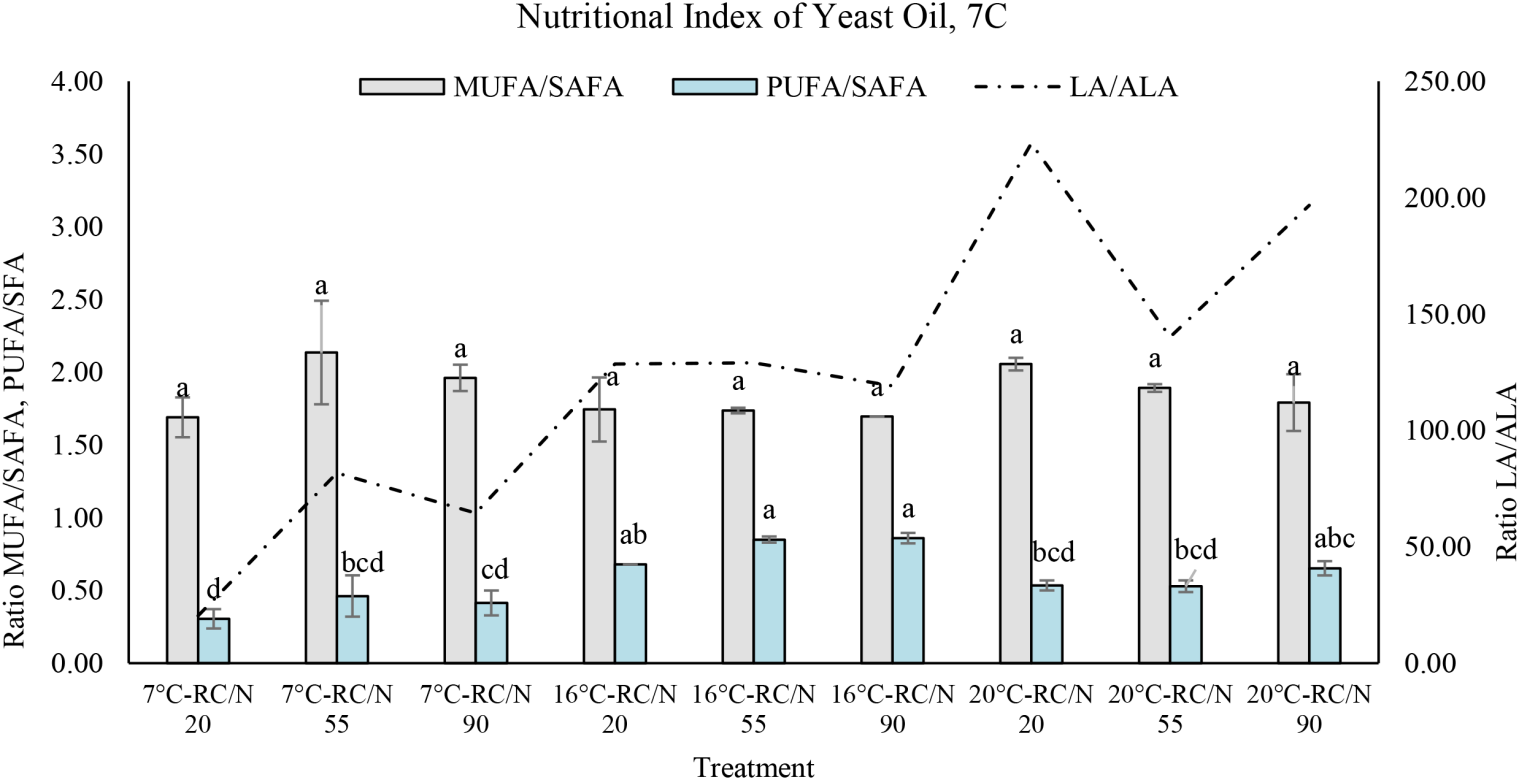
Nutritional indices of *S. gelidoterrea* 7C oil under different temperature conditions (thermal shift to 7°C, 16°C, or maintained at 25°C) and carbon/nitrogen (C/N) ratios of 20, 55, and 90. Shown are the MUFA/SAFA and PUFA/SAFA ratios (left y-axis), and the LA/ALA ratio (right y-axis). Different letters indicate statistically significant differences (p < 0.05).

The analysis of lipid ratios MUFA/SAFA, PUFA/SAFA, and LA/ALA of *S. gelidoterrea* revealed more stable trends, generally considered (**Figure 8**). The MUFA/SAFA ratio remained relatively stable across treatments (1.69–2.14), with slightly higher values observed at 20°C–C/N 20 (2.06 ± 0.04) and 7°C–C/N 55 (2.14 ± 0.36), suggesting a greater proportion of monounsaturated fatty acids under extreme or intermediate temperatures with limited nitrogen. However, no statistically significant differences were found among the conditions (p = 0.1642). In contrast, the PUFA/SAFA ratio showed a marked increase with temperature, peaking at 16°C–C/N 90 (0.86 ± 0.04), indicating that moderate conditions favor the accumulation of polyunsaturated fatty acids. The LA/ALA ratio exhibited the greatest variation among treatments, ranging from 20.38 (7°C–C/N 20) to 223.32 (20°C–C/N 20), with considerable standard deviations in certain conditions (e.g., 133.29 at 16°C–C/N 90), highlighting a high sensitivity of the omega-6/omega-3 balance to cultivation parameters.

## 5 Discussion

The aquaculture industry urgently requires alternative sources of nutrients, particularly rich in proteins and lipids, as traditional inputs like vegetable and fish oils face long-term sustainability limitations (Mahdy et al., 2022). This is driven by rising global demand and the environmental impact of current production systems. Identifying new sources is thus critical for future food security (Gasco et al., 2018). Microorganisms have emerged as promising candidates to diversify feed ingredients (Blomqvist et al., 2018), prompting research focused on their production (Poontawee and Limtong, 2020), scale-up (Sriphuttha et al., 2023), and *in vitro* and *in vivo* evaluation (Neuls et al., 2021; Xu et al., 2024; Filippucci et al., 2016). This study aimed to improve lipid production in two native yeast strains isolated from distinct natural environments. Strain 7C, isolated from volcanic soils of the Villarrica Volcano in southern Chile, was phylogenetically assigned to the species *S. gelidoterrea*. Phylogenetic reconstruction based on rRNA gene sequences revealed that strain 7C clusters robustly (bootstrap support 100/99) with type strains of *S. gelidoterrea*, such as CBS 15580_T,_ CGMCC2.4893, and CBS 9627. This tight clustering within the *gelidoterrea* clade supports its taxonomic placement and suggests a close evolutionary relationship among strains inhabiting cold and oligotrophic environments. Members of this clade have been primarily associated with alpine or glacial soils, indicating a shared ecological niche characterized by low temperatures and periodic nutrient limitations (Filippucci et al., 2016). Such environmental pressures may have driven the evolution of specific adaptive traits, including membrane remodeling via increased synthesis of polyunsaturated fatty acids (PUFAs), which enhance membrane fluidity under cold stress. These lipidomic adaptations are not only essential for maintaining cellular integrity in cold habitats but also present potential for biotechnological applications. In particular, cold-tolerant lipid-producing yeasts like *S. gelidoterrea* 7C could be exploited for the production of PUFA-rich biomass under low-energy cultivation conditions, with relevance to fish nutrition and sustainable feed development. *Rhodotorula mucilaginosa* has been extensively studied as an oleaginous yeast (Sun et al., 2020; Hu et al., 2022; Tsai et al., 2022; Rosas-Paz et al., 2024). Numerous studies have demonstrated its ability to accumulate high levels of intracellular lipids, particularly under nitrogen-limiting conditions. For instance, Li et al. (2022) reported lipid yields exceeding 60% of dry cell weight when cultivated in glucose-based media under nutrient stress. Similarly (Braunwald et al., 2013), emphasized the importance of the carbon-to-nitrogen (C/N) ratio as a key factor influencing microbial lipid synthesis. In addition (Sitepu et al., 2013), identified *R. mucilaginosa* as one of the most efficient and adaptable strains for industrial-scale fermentation processes. Nevertheless, even within well-characterized species such as *R. mucilaginosa*, native isolates can exhibit unique physiological traits or local adaptations derived from their specific environmental origins, making them attractive targets for further biotechnological investigation. Thus, the isolation and study of new local strains remains relevant for both basic and applied research. In this context, *S. gelidoterrea*, isolated in this study from volcanic soil, represents a particularly noteworthy example. Volcanic soils are often characterized by extreme physicochemical conditions, low nutrient availability, and high environmental variability, which may drive the development of distinctive metabolic adaptations. Although oleaginous properties have not been previously reported for this species, the physiological responses observed here, especially under nutrient-limited conditions, suggest that it could have relevant potential for microbial lipid production. Therefore, both species contribute meaningful value to the field: *R. mucilaginosa* as a well-established model that may exhibit additional properties due to its local origin, and *S. gelidoterrea* as a novel microbial resource with promising but unexplored capabilities.

In this context, evaluating the physiological and metabolic capacities of native strains is essential for accurately characterizing their biotechnological potential. The distinct positioning of BY4741 reflects a narrower metabolic profile and reduced phenotypic plasticity when compared to wild soil isolates. Specifically, strains 7C and Rho 6S, both recovered from soil environments, exhibited growth patterns more similar to each other than to BY4741. However, their divergence along PC1 and PC2 suggests that, despite sharing a general ecological origin, they may have evolved distinct adaptive strategies for carbon source utilization and stress tolerance. This divergence could be linked to the specific characteristics of their native habitats: strain 7C was isolated from volcanic soil, typically low in organic matter and marked by extreme physicochemical conditions, whereas Rho 6S originated from a soil richer in organic content, which may have favored a broader metabolic versatility.

Previous studies have reported that wild yeast isolates often possess broader metabolic capabilities and higher environmental robustness than laboratory strains (Liti et al., 2009; Warringer et al., 2011; Boynton and Greig, 2014). The divergence observed between 7C and Rho 6S further supports the notion of substantial phenotypic diversity even among wild strains from similar niches. Furthermore, physiological evaluations such as those performed in this study are instrumental in identifying specific metabolic capacities that can be harnessed for applied purposes. Compounds such as xylose and glycerol, frequently detected as substrates supporting growth in soil isolates, are of particular interest given their availability as by-products from agro-industrial processes (e.g., lignocellulosic hydrolysates and biodiesel production) (Moran-Mejía et al., 2022). The ability of certain wild yeast strains to efficiently metabolize these substrates not only highlights their metabolic versatility but also opens avenues for their use in sustainable bioprocesses. In the context of, these findings are especially relevant, as such compounds could be integrated into culture media for microbial biomass production or serve as feedstock in microbial fermentation systems aimed at generating high-value protein and lipids ingredients (Srirengaraj et al., 2023). The yeast strains evaluated in this study were isolated from soil, an environment where various fungal genera have demonstrated relevant metabolic capacities, including lipid accumulation (Díaz et al., 2017; Díaz-Navarrete et al., 2023). In particular, *S. gelidoterrea* strain 7C has been scarcely reported in the literature (Park and Srinivasan, 2024), and to date, no studies have documented its potential as an oleaginous yeast. However, previous research on related species within the same genus allows for useful comparisons. In this sense, Filippucci et al. (2016) evaluated various oleaginous yeasts, including *S. aeria* (formerly *Cryptococcus aerius*) and *S. terricola* (formerly *Cryptococcus terricola*), demonstrating that both the carbon source and cultivation temperature significantly influence lipid yield and fatty acid composition. In this sense, Filippucci et al. (2016) evaluated various oleaginous yeasts, including *S. aeria* (formerly *Cryptococcus aerius*) and *S. terricola* (formerly *Cryptococcus terricola*), demonstrating that both the carbon source and cultivation temperature significantly influence lipid yield and fatty acid composition. For example, *S. terricola* grown on xylose yielded 44% saturated fatty acids, while sucrose resulted in 59.02% unsaturated fatty acids. In our study, *S. gelidoterrea* 7C cultivated at 20°C with a cold shock at 7°C (after 72 h) under a C/N ratio of 20 and using glucose as the carbon source, exhibited a lipid profile composed of 33 ± 0.78% saturated fatty acids, 56 ± 3.24% monounsaturated fatty acids (predominantly oleic acid, C18:1ω9), and 10% polyunsaturated fatty acids (PUFAs). Among the latter, high-value compounds such as arachidonic acid (ARA, C20:4 ω6, 0.33%), docosahexaenoic acid (DHA, C22:6 n3, 0.52%), eicosapentaenoic acid (EPA, C20:5 ω3, 0.45%), and linoleic acid (C18:2 ω6c, 9%) were identified. These results suggest that the temperature shift from 20°C to 7°C may trigger PUFA biosynthesis, a common adaptive mechanism in psychrophilic or psychrotolerant yeasts, enhancing membrane fluidity under cold stress. Aiello et al. (2021) reported that *S. terricola*, cultivated in batch and fed-batch modes using lignocellulosic hydrolysates, achieved 13.81 g/L lipid content with 67% unsaturated fatty acids, highlighting the effectiveness of fed-batch strategy to enhance lipid biosynthesis. Similarly, Tasselli et al. (2019) optimized enzymatic hydrolysis of driftwood for lipid production by *S. terricola* using RSM, identifying optimal conditions that predicted a lipid accumulation of 27.32%, experimentally validated at 25.26%, with over 70% oleic acid content. In our study, also using RSM, *S. gelidoterrea* under cold shock conditions (7°C), regardless of the C/N ratio, reached an oleic acid content of 57.41 ± 1.71% of total lipids. *R. mucilaginosa* is a yeast that has been documented not only as oleaginous but also for other potentials, including its use as a protein-rich yeast, a source of pigments, and various functional (Li et al., 2022; Sahay et al., 2022).

Abaza et al. (2024) evaluated the effect of high glucose concentrations on the biosynthesis of long-chain polyunsaturated fatty acids (LC-PUFA) in *R. mucilaginosa*, demonstrating that, through a two-stage cultivation system, this yeast can accumulate over 20% of its dry cell weight as lipids. In our study, the Rho 6S strain exhibited a remarkable lipid profile, characterized by a high content of unsaturated fatty acids, averaging 28% ± 4.9 LC-PUFA, 53.95% ± 4.64 MUFA, and less than 20% SAFA. The thermal shock condition at 16°C with a C/N ratio of 20 promoted PUFA production, particularly linoleic acid (C18:2 ω6c) at 27%, linolenic acid (C18:3 ω3) at 4.85%, arachidonic acid (ARA, C20:4 ω6) at 0.3%, and docosahexaenoic acid (DHA, C22:6 ω3) at 0.12%. Conversely, cultivation at 7°C favored MUFA production, reaching 58.85% oleic acid (C18:1 ω9c). Several studies have confirmed that *R. mucilaginosa* exhibits a promising lipid profile for biotechnological and nutritional applications, with total lipid accumulation reaching up to 46.7% w/w under specific conditions such as high C/N ratios (≥65) and reduced temperatures (15°C) (Li et al., 2022). The predominant fatty acid fractions include MUFA, especially oleic acid (C18:1), with values exceeding 60%, followed by SAFA such as palmitic (C16:0) and stearic (C18:0) acids, and to a lesser extent PUFA, mainly linoleic acid (LA, C18:2) and α-linolenic acid (ALA, C18:3) (Sitepu et al., 2013; Díaz-Navarrete et al., 2024). These lipid profiles result in favorable nutritional indices, with PUFA/SAFA ratios ranging from 0.5 to 0.9, ω6/ω3 ratios below 4:1, and LA/ALA ratios between 3.7 and 6.3, depending on cultivation conditions. These parameters reflect a healthy lipid profile, comparable to high-quality vegetable oils, positioning *R. mucilaginosa* as a viable source of functional lipids for applications in human nutrition, aquaculture, and nutraceutical formulations. According to our results, the oil produced by *R. mucilaginosa* Rho 6S showed a balanced lipid profile under various cultivation conditions. The MUFA/SAFA ratio ranged from 2.57 to 3.61, indicating a significant presence of monounsaturated fatty acids, comparable to high-quality oils such as olive oil (Bortoluzzi et al., 2025). The PUFA/SAFA ratio ranged from 1.07 to 2.13, suggesting an adequate PUFA content that contributes to nutritional benefits without severely compromising oxidative stability. Notably, the LA/ALA ratio varied from 4.31 to 12.76, with several experimental conditions falling below the recommended threshold (<6:1) for an appropriate ω6/ω3 balance. These findings further support the potential of *Rhodotorula*-derived oil for functional applications in the food and nutraceutical industries. In contrast, the lipid profile of *S. gelidoterrea* strain 7C exhibited nutritional and technological characteristics that differ from those of conventional oils. The MUFA/SAFA ratio, ranging from 1.69 to 2.14, indicates a moderately high MUFA content, which enhances the oil’s thermal and oxidative stability. However, the PUFA/SAFA ratio was below 0.86, reflecting a relatively low PUFA proportion. A particularly relevant finding is the high LA/ALA ratio, which ranged from 20.38 to 223.32, greatly exceeding the nutritionally recommended upper limit (<6:1). Although this imbalance does not appear to significantly affect fish growth, it may influence lipid metabolism and fatty acid deposition in tissues (Paulino et al., 2018; Rincón-Cervera et al., 2020). From a human nutritional perspective, this disproportion could limit the health-promoting potential of the oil. Furthermore, the proportions of MUFA, PUFA, and, particularly, of ω3 and ω6 fatty acids (Duman-Özdamar et al., 2022) are key determinants of fish nutritional quality (Colombo et al., 2018; He et al., 2022), affecting both tissue fatty acid composition and health outcomes for consumers (Azcuy et al., 2025).

The detailed characterization of nutritional indices, such as MUFA/SAFA, PUFA/SAFA, ω3/ω6, and LA/ALA, in oleaginous yeasts holds strategic significance (Emam et al., 2020), as these microorganisms are emerging as some of the most promising lipid sources for future applications (Småros et al., 2025). In our study, *S. gelidoterrea* exhibited remarkable physiological resilience, maintaining high lipid accumulation even under nitrogen-restricted and low-temperature conditions. This behavior was reflected in the increased levels of mono-unsaturated fatty acids (MUFA), particularly oleic acid, at 7°C and a C/N ratio of 55, as well as in its unusual capacity to synthesize long-chain polyunsaturated fatty acids (LC-PUFA) such as arachidonic acid (ARA), eicosapentaenoic acid (EPA), and docosahexaenoic acid (DHA) under C/N 90 at 16°C. These features suggest the involvement of specific regulatory mechanisms, including the activation of enzymes such as Δ9 desaturase (OLE1) and acetyl-CoA carboxylase (ACC1), which are known to respond to cold stress and nitrogen limitation (Li et al., 2023). It is worth noting that essential structural lipids in yeast include phospholipids such as phosphatidylcholine (PC), phosphatidylethanolamine (PE), phosphatidylinositol (PI), phosphatidylserine (PS), and phosphatidic acid (PA), which are primarily composed of fatty acids like palmitic, oleic, and linoleic acids, whose proportion and degree of unsaturation determine membrane fluidity and functionality (Řezanka et al., 2016). This adaptive response may be linked to the ecological origin of S. gelidoterrea, isolated from cold, nutrient-poor volcanic soils, where natural selection may have favored energy conservation strategies based on structurally and functionally efficient unsaturated lipids (Rossi et al., 2009). Recent studies have shown that cold stress induces lipid remodeling in psychrotolerant yeasts, including increased fatty acid unsaturation and changes in phospholipid and sterol composition, such as ergosterol, which enhance membrane stability under thermally adverse conditions (Turchetti et al., 2025). Unlike *R. mucilaginosa*, which exhibits reduced lipid accumulation under high C/N ratios, *S. gelidoterrea* displays a metabolic strategy that optimizes carbon partitioning toward highly functional lipids. These findings not only reveal a notable physiological novelty but also highlight the biotechnological potential of this yeast for industrial applications under extreme environmental conditions.

In recent years, plant-derived dietary lipids have gained increasing nutritional relevance in salmon aquaculture (Menoyo et al., 2007). This trend is driven largely by the implementation of high-energy feed formulations and the growing recognition of the metabolic roles played by saturated and monounsaturated fatty acids in both animals and humans (Chen and Liu, 2020). Traditionally, fish oil has been considered the benchmark ingredient in aquafeed formulations due to its balanced fatty acid profile (Dubois et al., 2007; Geurden et al., 2009; Turchini et al., 2011). However, most vegetable oils and their blends fail to fully replicate this profile, particularly in terms of n-6/n-3, SAFA/MUFA, PUFA/SAFA, and LA/LNA ratios. The substitution of fish oil with vegetable oils in salmonid diets has proven effective in sustaining growth performance. However, a critical challenge lies in preserving an appropriate n-6/n-3 ratio, as imbalances in this proportion have been linked to adverse effects on growth, inflammation, antioxidant capacity, and nutrient metabolism in fish (Tan et al., 2009; Zuo et al., 2015). Commonly used vegetable oils in aquafeeds, such as canola and soybean oils, typically exhibit n-6/n-3 ratios ranging from 2.2 to 6.7, respectively (Dubois et al., 2007), substantially higher than those found in fish oil. Freshwater fish species possess the enzymatic capability to elongate and desaturate short-chain fatty acids, such as α-linolenic acid (18:3n-3; LNA) and linoleic acid (18:2n-6; LA), into long-chain polyunsaturated fatty acids (Paulino et al., 2018). In this regard, (Hamad et al., 2024) demonstrated that rainbow trout can efficiently synthesize docosahexaenoic acid (22:6n-3; DHA) from LNA when fed diets with n-6/n-3 ratios between 1.0 and 2.0, even in the absence of fish oil, achieving growth and lipid deposition levels comparable to those fed fish oil-based diets. These findings are consistent with (Colombo et al., 2018), who observed enhanced DHA deposition in Atlantic salmon muscle when the dietary LA/LNA ratio approached 1. Notably, the oleaginous yeast *R. mucilaginosa* (Rho 6S) consistently exhibits an LA/LNA ratio close to 1 under various culture conditions, including different temperatures and C:N ratios, which is considered optimal from a nutritional standpoint for fish. In contrast, *S. gelidoterrea* (7C) achieves a similar ratio only at 7°C. For comparison, conventional vegetable oils used in aquafeeds, such as canola and soybean oil, present LA/LNA ratios ranging from 2.2 to 7.8 (Dubois et al., 2007), highlighting the potential of these yeast strains as alternative lipid sources in aquafeed formulations. Several studies have shown that n-3 polyunsaturated fatty acids (PUFA) are more digestible than their n-6 counterparts (Eroldoǧan et al., 2013). Therefore, dietary n-3/n-6 ratios can significantly influence lipid digestibility, energy utilization immune function, and muscle lipid deposition (Colombo et al., 2018). Additionally, diets with lower saturated fatty acid (SAFA) content tend to show greater overall lipid digestibility compared to those with higher SAFA levels (Menoyo et al., 2007). Sissener et al. (2025) further reported that dietary SAFA levels exceeding 15% did not impair growth performance or fillet quality in Atlantic salmon. In this context, the oleaginous yeasts *R. mucilaginosa* and *S. gelidoterrea* emerge as novel biotechnological ingredients of interest. Both species exhibit SAFA contents around 15% and PUFA/SAFA ratios close to or below 1 under specific cultivation conditions. This lipid profile more closely resembles that of fish oil than that of most vegetable oils, which are typically rich in n-6 fatty acids and relatively deficient in n-3 (Dubois et al., 2007), potentially enhancing the nutritional and metabolic utilization of dietary lipids in aquaculture species.

From a nutritional perspective, the PUFA/SAFA ratio is a key indicator of the health quality of dietary fats. The oleaginous yeasts evaluated in this study exhibited a ratio around 0.86, indicating a favorable and balanced fatty acid composition. This lipid profile is associated with potential cardiovascular benefits due to the presence of polyunsaturated fatty acids in adequate proportions. Compared to conventional plant-based oils, these yeasts offer a promising alternative. Their composition supports their application in health-oriented foods and sustainable aquafeeds.

## 6 Conclusion

This study highlights the remarkable biotechnological potential of two native yeasts, *R. mucilaginosa* Rho 6S and *S. gelidoterrea* 7C, as sustainable lipid sources for applications in aquaculture, food, and other nutrition-related industries. R. mucilaginosa exhibited a well-balanced lipid profile, with MUFA/SAFA ratios (2.57–3.61), PUFA/SAFA (1.07–2.13), and LA/ALA (<6:1), corresponding to a high-quality nutritional oil. In contrast, S. gelidoterrea, despite displaying PUFA/SAFA ratios below 0.86 and LA/ALA values exceeding 20, demonstrated exceptional physiological resilience, maintaining elevated lipid accumulation under nitrogen-limited and low-temperature conditions. Notably, this species showed increased oleic acid levels at 7°C under a C/N ratio of 55, and an uncommon ability to synthesize long-chain polyunsaturated fatty acids (LC-PUFAs) such as ARA, EPA, and DHA under a C/N ratio of 90 at 16°C. From a nutritional perspective, these features are particularly valuable, as they enable the production of functional lipids with potential cardiovascular and metabolic health benefits. This study also provides the first lipid profile characterization of *S. gelidoterrea*, significantly expanding current knowledge on this species and supporting its development as a promising microbial platform for the production of high-value oils. Altogether, these findings reinforce the importance of exploring native yeasts as novel lipid-producing biofactories capable of generating advanced ingredients for functional foods and aquafeeds under controlled, sustainable, and stress-adaptive production systems.

## Data Availability

The original contributions presented in the study are publicly available. This data can be found here: NCBI GenBank, accession PV784691.
