# Supplementary material for: Native oleaginous yeasts Rhodotorula mucilaginosa and Solicoccozyma gelidoterrea: a sustainable biotechnological alternative for lipid production with potential application in diets for farmed fish
Source: Front Fungal Biol. 2026 Feb 10;7:1664434. doi: 10.3389/ffunb.2026.1664434 (PMC12929513; doi:10.3389/ffunb.2026.1664434)
Supplement: Supplementary file 1 [file Table1.docx]

SUPLEMENTARY MATERIALES

Table S1. Calculated µmax from growth curves in differents culture media.

*Caffeine, CuSO4,DTT, EtOH 8, EtOH 9, EtOH_10, Fructose ,G418, Galactose, Glycerol, 2 H2O2, KCl ,Lactose, MaltoDextrine, Maltose, Methanol 8, NaCl, p-coumaric, Raffinose, Sucrose, SDS, Sorbiltol, Xylose.

Table S2. Lipid profile *R. mucilaginosa*

Table S3. Lipid profile *S. gelidoterrea*

*Table S4. Descriptive statistics of Rhodotorula sp. strain under different temperatures (25 °C, 16 °C, 7 °C) and C:N ratios (20, 55, 90).*


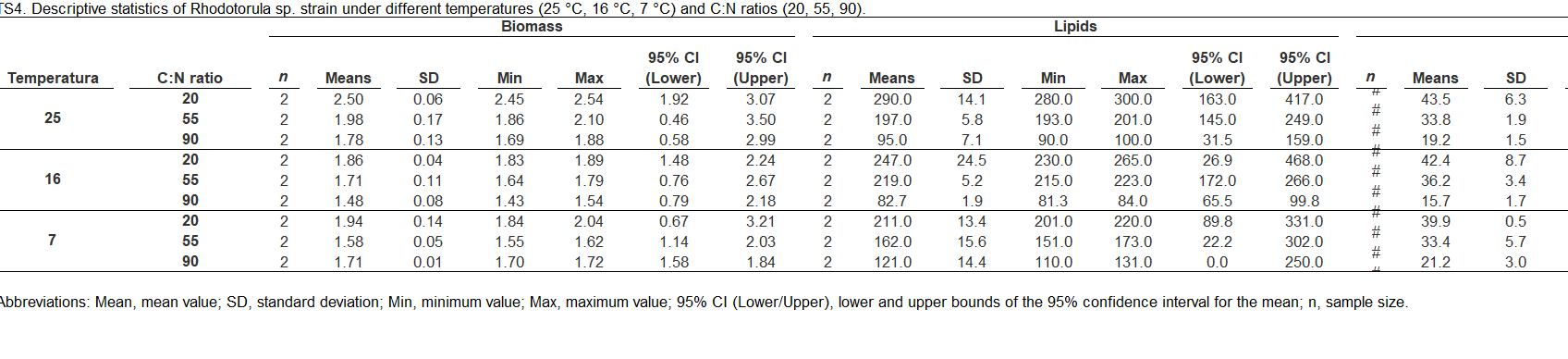

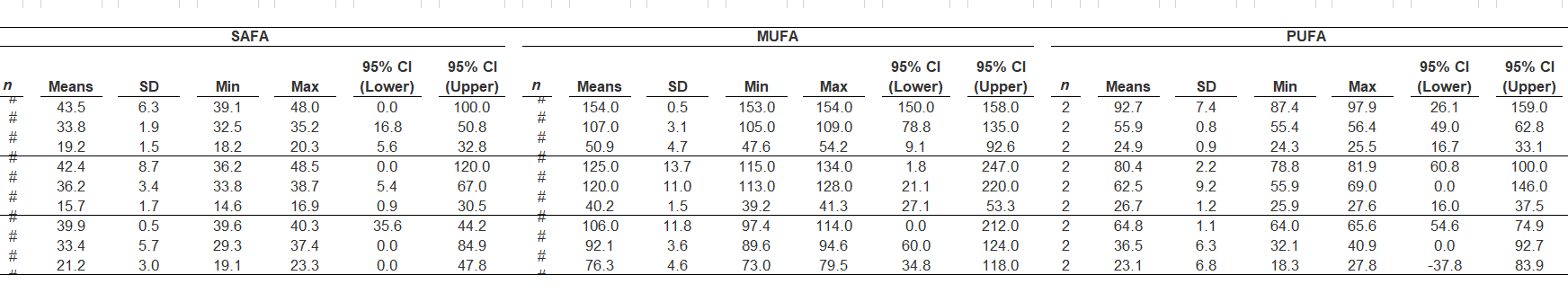

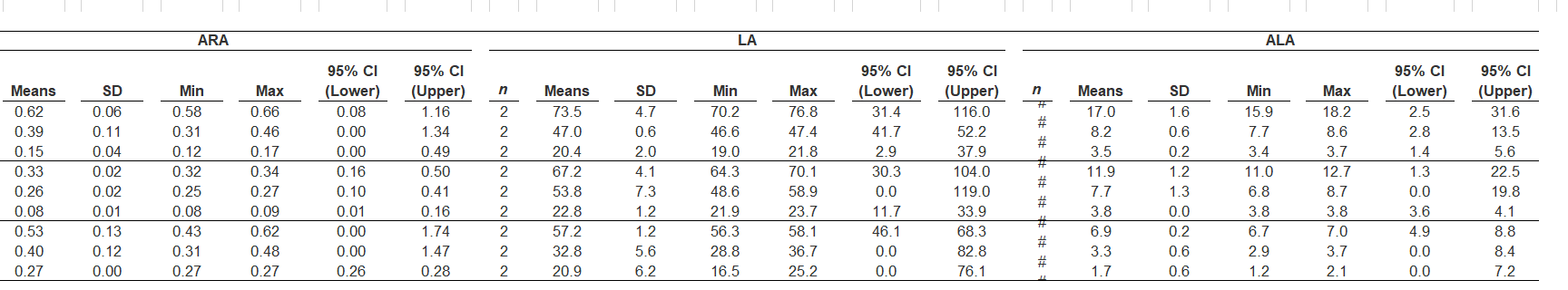


*
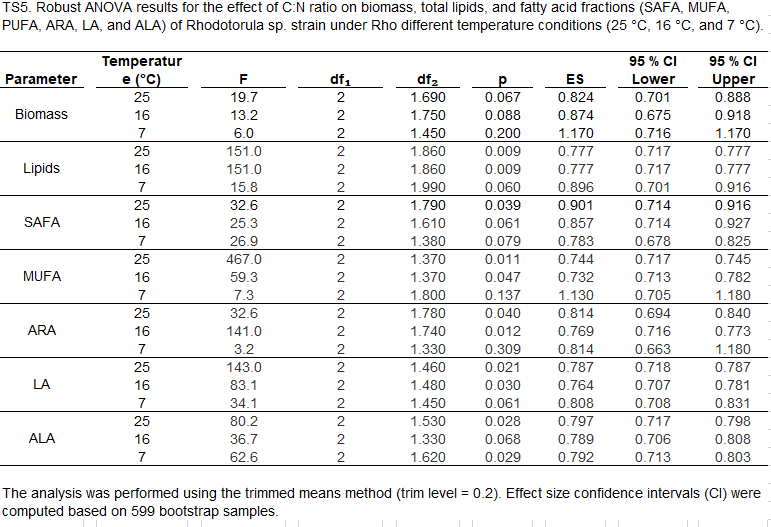
*

*Table S6. Descriptive statistics of Solicoccozyma sp. strain 7C under different temperatures (25 °C, 16 °C, 7 °C) and C:N ratios (20, 55, 90).*

*
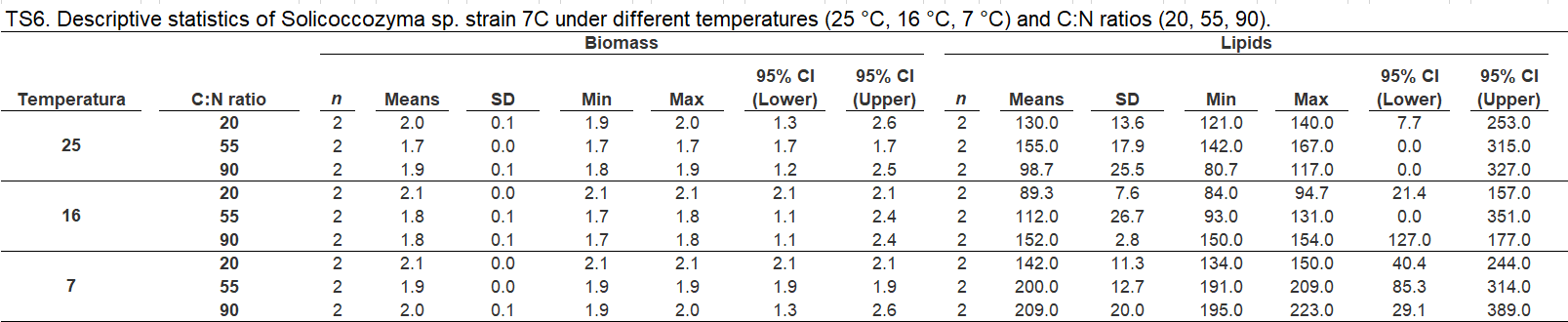
*

*
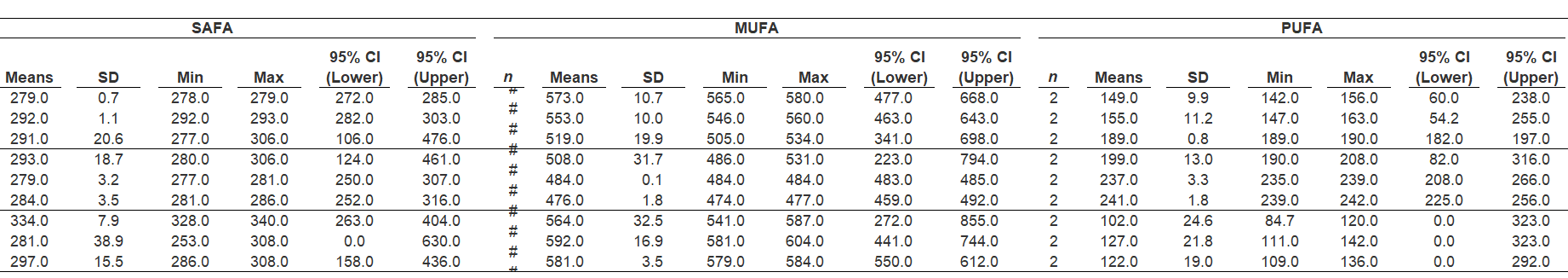
*

*
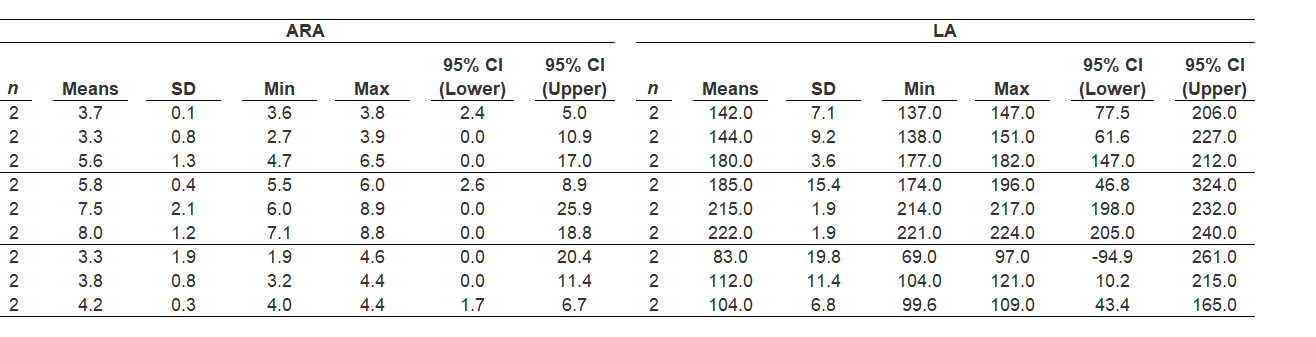
*

*
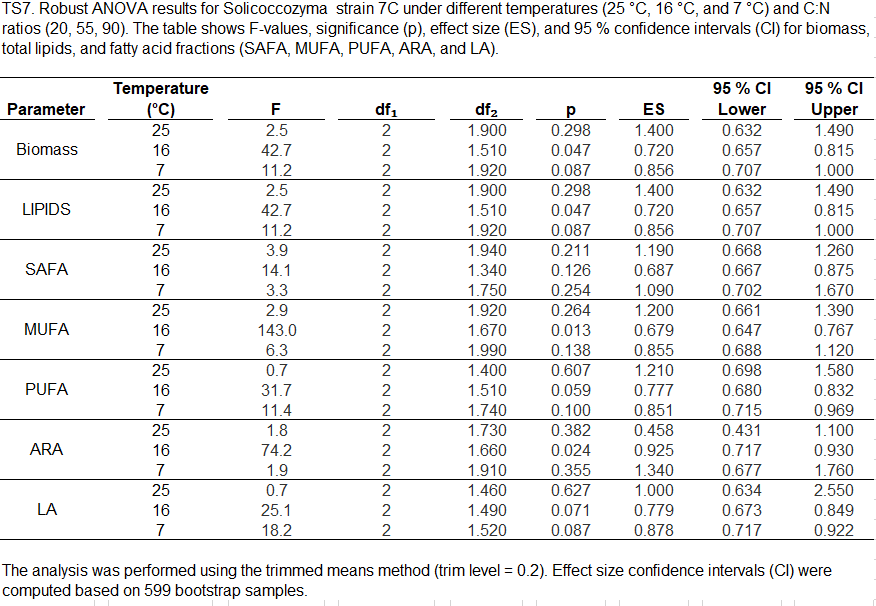
*

*
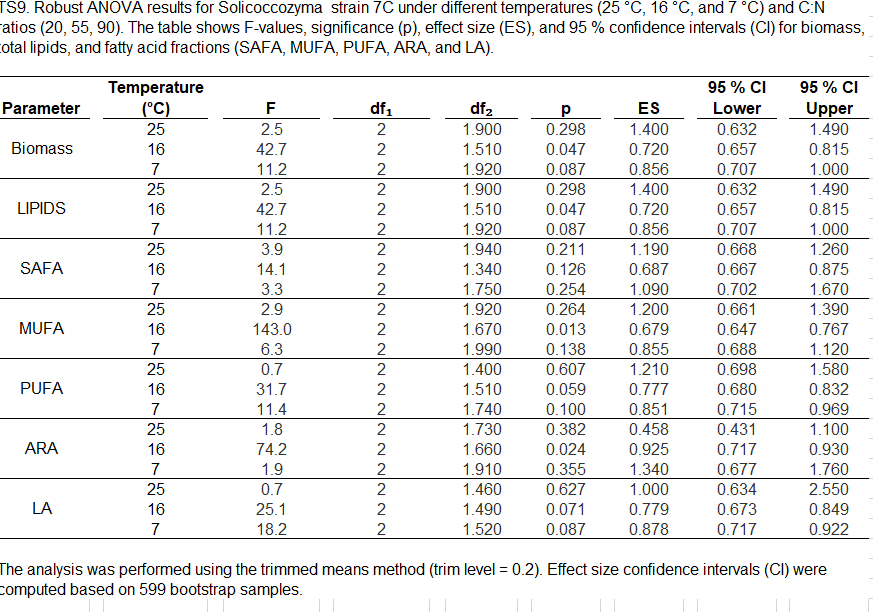
*
